# Molecular Mechanisms Driving Larval Green Color Adaptation in Swallowtail Butterfly, *Papilio memnon*

**DOI:** 10.1093/gbe/evag253

**Published:** 2026-10-13

**Authors:** Liang Liu, Shinya Komata, Kai Wu, Takehiko Itoh, Tetsuya Kojima, Haruhiko Fujiwara

**Affiliations:** Department of Integrated Biosciences, Graduate School of Frontier Sciences, The University of Tokyo, Kashiwa, Japan; Helsinki Institute of Life Science, University of Helsinki, Helsinki, Finland; School of Life Science and Technology, Institute of Science Tokyo, Tokyo, Japan; College of Life Sciences, Shangrao Normal University, Shangrao, China; School of Life Science and Technology, Institute of Science Tokyo, Tokyo, Japan; Department of Integrated Biosciences, Graduate School of Frontier Sciences, The University of Tokyo, Kashiwa, Japan; Department of Integrated Biosciences, Graduate School of Frontier Sciences, The University of Tokyo, Kashiwa, Japan

**Keywords:** molecular evolution, gene regulation, pigmentation, larval coloration, swallowtail butterfly

## Abstract

Insects have evolved diverse anti-predator strategies, with coloration playing a central role through mechanisms such as mimicry and camouflage. Most swallowtail butterfly larvae switch from a black/white bird-dropping mimetic pattern to a green camouflage pattern at the final (5th) instar. In *Papilio memnon*, this switch from mimicry-like to camouflage also occurs at the final instar. But *P*. *memnon* initiates accumulating green coloration at the 3rd instar, earlier than its closely related species such as *Papilio xuthus* and *Papilio polytes*. This interspecific variation in the timing of green coloration provides a great model for understanding evolutionary and developmental mechanisms that drive the diversification of insect body coloration among closely related species. Through in vivo electroporation-mediated RNA interference (RNAi), we confirmed some *bilin-binding proteins* (*BBP*s), and *juvenile hormone-binding proteins* (*JHBP*s) as key effectors of green pigmentation in *P. memnon*. Further genomic analysis revealed that these *BBP* and *JHBP* genes are organized into conserved tandem clusters with the same gene order and transcriptional orientation across *Papilio* species. All *BBP*s and certain *JHBP*s within the corresponding clusters displayed markedly elevated expression in early-stage *P. memnon* larvae (green) relative to *P. xuthus* larvae (black). Together, our results suggest that heterochronic expression of conserved *BBP* and *JHBP* gene clusters contributes to the earlier onset of green coloration in *P. memnon*, potentially through evolutionary changes in upstream regulatory mechanisms shared by these genes.

Significance Closely related species can differ strikingly in the developmental timing of body coloration, yet the genetic mechanisms underlying such heterochronic shifts remain poorly understood. Here, we show that the swallowtail butterfly (*Papilio memnon*) evolved an earlier onset of larval green color through precocious expression of tandem clusters of *bilin-binding protein* and *juvenile hormone-binding protein* genes. Functional knockdown confirms that specific members of these clusters contribute to the blue and yellow components of larval green coloration, whereas their genomic organization is highly conserved across Papilio species. These findings suggest that heterochronic regulatory divergence, rather than changes in gene content, can drive the evolution of adaptive coloration and illustrate how shifts in developmental timing contribute to phenotypic diversification.

## Introduction

Insects have evolved a variety of adaptive traits to mitigate predation pressure. Among these traits, coloration plays a critical role: some patterns function as aposematic signals to warn predators of toxicity or unpalatability, while others facilitate crypsis by enabling organisms to blend into their surroundings (Bates 1862; Müller 1879; Wallace 1889; Caro and Koneru 2021; Robinson et al. 2023). These strategies are well documented in caterpillars of Lepidoptera, such as the *Papilio* genus, whose larvae are relatively immobile and highly vulnerable to predation by birds and other visual predators (Jin et al. 2019). To reduce the predation risk, many *Papilio* caterpillars have evolved visually deceptive color patterns such as mimicry and camouflage (Pasteur 1982; Futahashi and Fujiwara 2008). The larvae of *Papilio* butterflies display a striking ontogenetic color pattern shift: from bird-dropping mimicry in early instars to cryptic green camouflage in the final (5th) instar (Futahashi and Fujiwara 2008). Specifically, the 1st to the 4th instar larvae shows a black and brown color pattern with a white V-shaped pattern resembling bird droppings (Fig. 1a). By contrast, the 5th instar larvae display a green body with characteristic false eyespots on the third thoracic segment (T3) and oblique white, black, or dark brown bands across the abdominal segments, which form a V-shaped pattern when viewed dorsally (Fig. 1a). This dramatic color pattern transition is attributed to a rapid decrease in juvenile hormone (JH) titers at the beginning of the 4th instar, immediately following the 3rd molt (Futahashi and Fujiwara 2008). The critical window for the hormonal modulation is termed the juvenile hormone-sensitive period (JHSP), defined as the approximately 12-hour period post-molt during which patterning cues are established (Fig. 1b) (Futahashi and Fujiwara 2008; Jin et al. 2019).

**Fig. 1.**
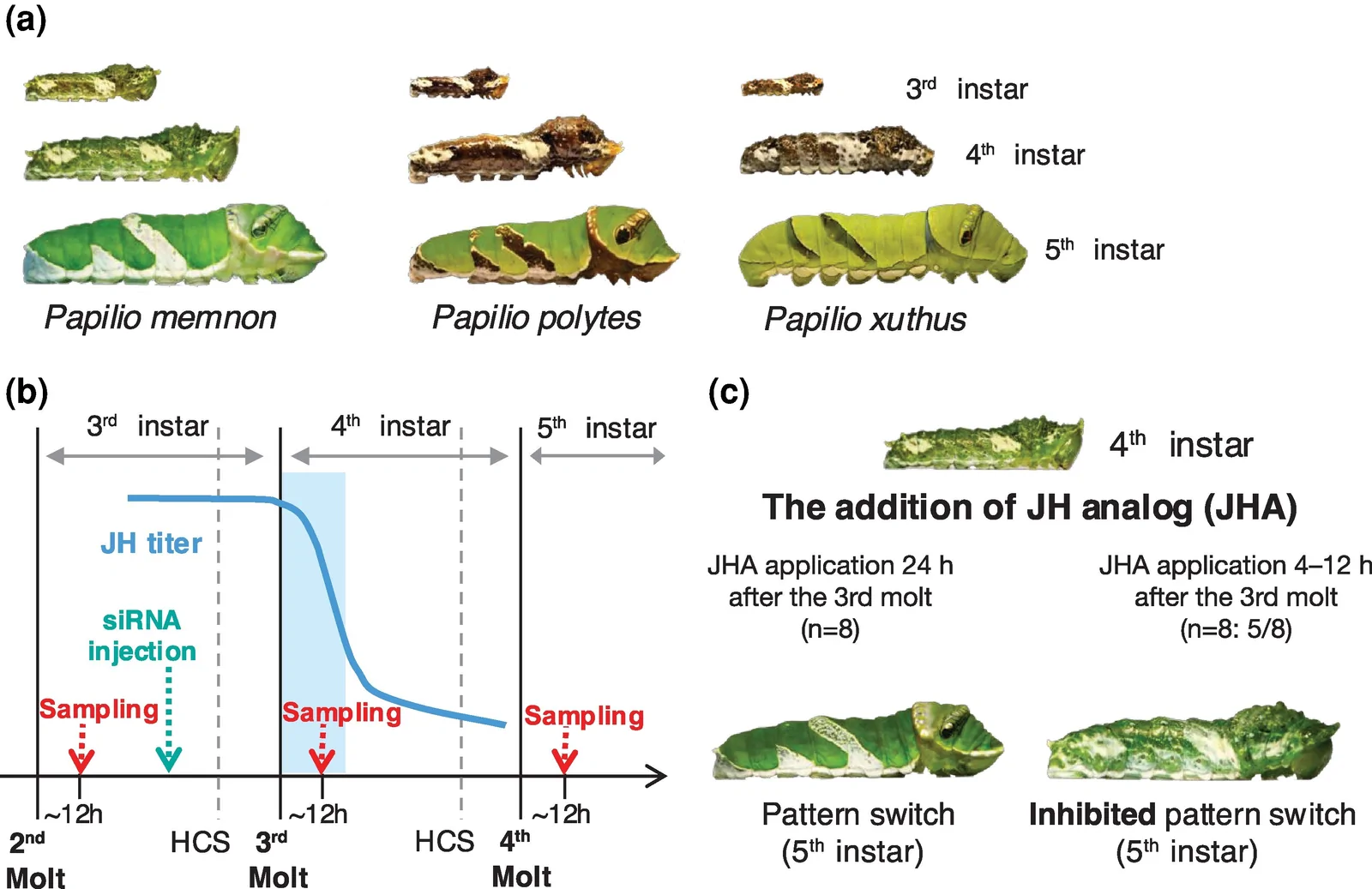
Larval color patterns of *Papilio memnon*, *Papilio polytes* and *Papilio xuthus*, and the effects of juvenile hormone (JH). a) Phenotypes of 3rd, 4th and 5th instar larvae in *P*. *memnon*, *P*. *polytes* and *P*. *xuthus*. All three species switch from a bird dropping mimicry to a camouflage pattern when they molt from the 4th to 5th instar. Unlike *P. polytes* and *P. xuthus*, *P*. *memnon* exhibits a green body coloration from the 3rd instar onward. b) Expected JH titer in *Papilio* caterpillars adapted from Futahashi and Fujiwara (2008). The shaded region indicates the JH-sensitive period (JHSP, the first 12 h after the third molt) when JH levels drop rapidly. HCS: head capsule slippage. Epidermal samples were collected for RNA-seq at 12 h post-molt. siRNA was injected before HCS during the 3rd instar. c) JH analog (fenoxycarb) treatment. Application of the JH analog during the JHSP (4 to 12 h after the third molt) in *P. memnon* prevented the color pattern switch, producing 5th instar larvae that retained the 4th-instar phenotype.

The larval color pattern switch is hypothesized to comprise two independent processes: (i) a prepatterning process during the JHSP, in which segment-specific pattern boundaries are established and (ii) a pigmentation phase during the molting period, where pigment synthesis and deposition occur within prepatterned regions (Jin et al. 2019; Jin et al. 2020). A recent study in *P. xuthus* identified a more specific JH-dependent network involving three homeobox genes, *clawless* (*cll*), *abdominal-A* (*abd-A*), and *Abdominal-B* (*Abd-B*) as key regulators of the prepatterning process (Jin et al. 2019). *cll* is involved in eyespot formation on the segment T3 of the 5th instar larvae, while *abd-A* and *Abd-B* contribute to the development of the V-shaped marking on posterior abdominal segments (Jin et al. 2019). On the other hand, the pigmentation process relies on well-characterized pigment synthesis genes (Shirataki et al. 2010; Futahashi et al. 2012). Melanin-related genes such as *Tyrosine hydroxylase* (*TH*), *dopa decarboxylase* (*DDC*), *yellow*, *tan*, and *laccase 2* are associated with the larval cuticular black pigmentation (Futahashi and Fujiwara 2005, 2007; Futahashi et al. 2010; Shirataki et al. 2010). Another melanin-related gene, *ebony,* which converts dopamine into N-β-alanyl-dopamine (NBAD), is responsible for the red coloration in the eyespots of *P*. *xuthus* (Shirataki et al. 2010; Li et al. 2015).

In addition to various melanin-based dark colors, *Papilio* larvae also show a green body color, which serves as cryptic camouflage to hide the caterpillars in the host plants (Tullberg et al. 2005). In *P*. *xuthus*, the expression of pigmentation genes associated with green coloration is thought to be initiated in JHSP, and it has been hypothesized that JH titer regulates the pigmentation gene expression (Futahashi and Fujiwara 2008; Jin et al. 2019), but the relationship between green pigmentation genes and JH titer is not fully understood. Green larval coloration in Lepidopteran species typically results from a combination of bilin pigment (blue) and carotenoids (yellow) (Riley et al. 1984; Shirataki et al. 2010; Futahashi et al. 2012). It has been shown that the spatial expression of *bilin-binding protein*s (*BBP*s) and *carotenoid-binding protein 1* and *2* (*CBP1* and *CBP2*) or yellow-related genes in *P*. *xuthus* and *P*. *polytes* colocalized with the larval blue and yellow coloration, respectively (Shirataki et al. 2010; Futahashi et al. 2012). Additionally, a previous study suggested that larval and pupal protective green color of *P*. *polytes* resulted from the functions of *BBP*s contributing to the blue coloration and *juvenile hormone-binding protein*s (*JHBP*s) responsible for the yellow coloration (Yoda et al. 2020). *BBP*s belong to the lipocalin gene family, with tandemly arranged copies (*BBP1-6*) in both *P. polytes* and *P. xuthus* (Yoda et al. 2020). Species in the *Papilio* genus typically produce green pupae that serve as camouflage, and *BBP1* and *BBP2* have been shown to contribute to the formation of blue pigmentation within this green coloration (Yoda et al. 2020). For the yellow component, expression of *carotenoid-binding proteins* (*CBP1* and *CBP2*), both of which are members of the juvenile hormone-binding protein (*JHBP*) gene family, spatially colocalizes with yellow-pigmented regions in *P. xuthus* and were therefore considered possibly involved in yellow pigmentation (Futahashi et al. 2012). In *P. polytes*, knockdown of the *CBP1* homolog in the green areas of fifth-instar larvae led to a slight bluish change (due to the loss of yellow pigment; Yoda et al. 2020). However, knockdown of *CBP1* did not produce a discernible bluish shift in pupae of *P. polytes* (Yoda et al. 2020). These contrasting results suggest that the contribution of *CBP1* to pigmentation may differ across developmental stages. Moreover, these *JHBP*s, including *CBP1* and *CBP2*, form tandem gene clusters that contain six *JHBP*s (two *CBP*s) in *P. polytes* and nine in *P. xuthus* (Yoda et al. 2020). The specific roles of these gene clusters in blue and yellow pigmentation require further investigation, as certain genes may act redundantly or show stage-specific functions.

In *P*. *memnon*, 1st and 2nd instar larvae exhibit the black-and-white bird-dropping pattern as in *P*. *xuthus* and *P*. *polytes*. But unlike *P. xuthus* and *P. polytes* which retain black/brown body color till the final (5th) instar, *P. memnon* initiates green pigmentation earlier, with larvae already exhibiting a greenish body during the 3rd onward (Fig. 1a). Nevertheless, the overall larval pattern switch still occurs between the 4th and 5th instars, as in other *Papilio* species. Specifically, the pattern shifts from green with a white band to a green background featuring false eyespots and a white V-shaped marking between 4th and 5th instars in *P*. *memnon* (Fig. 1a). This variation in developmental timing of green coloration suggests a heterochronic change in pigment regulation and may reflect an adaptive evolutionary response to selective pressures. One potential driver of this diversification in body coloration is body size. *P*. *memnon* larvae are generally much larger than those of *P*. *xuthus*, as reflected in pupal mass: *P*. memnon pupae typically weigh from 1.5 to 3.5 g, about twice as much as *P*. *xuthus* (0.5 to 1.5 g) (Komata and Sota 2017; Komata et al. 2018). While the direct link between body size and the coloration of mimetic pattern remains to be tested, the effectiveness of bird-dropping mimicry may diminishe as *P. memnon* larvae increase in body size from the 3rd instar onward. In this context, greater visual detectability by predators due to larger body size may increase predation risk, thereby favoring an earlier onset of green pigmentation to enhance cryptic camouflage in *P. memnon*. Despite the potential significance, the genetic basis underlying this early onset of green pigmentation remains unknown. This interspecific variation presents a great model for understanding the evolutionary and developmental mechanisms underlying the diversification of larval body coloration in closely related species. In *P. xuthus* and *P. polytes*, genes implicated in green pigmentation, specifically *BBP*s and *JHBP*s, are arranged in tandem clusters (Yoda et al. 2020). Furthermore, gene family expansions with copy number variation have been widely associated with the evolution of adaptive traits across taxa (Fischer et al. 2008; Engsontia et al. 2014). This raises the possibility that *P. memnon* may have achieved the heterochronic shift in green pigmentation through changes in cluster composition, copy number, or regulation. Therefore, we asked: (i) Do the *BBP* and *JHBP* genes in *P. memnon* form conserved tandem clusters similar to those in *P. xuthus* and *P. polytes*, and if so, are they conserved in terms of copy number, gene order, and composition, or has species-specific diversification occurred?; and (ii) Do interspecific differences in the spatial and temporal expression dynamics of these cluster genes, or divergence in their functional roles, underlie the earlier onset of green coloration in *P. memnon*?

To address these questions, we first applied the juvenile hormone (JH) analog to examine whether a decline in JH titer, which regulates the shift from mimetic to camouflage patterns in *P. xuthus* (Futahashi and Fujiwara 2008), similarly controls the timing of color pattern transitions in *P. memnon*. To better evaluate the genomic architecture and evolutionary conservation of the green-related genes, we reannotated the genomes of three *Papilio* species (*P. memnon*, *P. polytes*, *P. xuthus*), focusing on the structural organization of *BBP* and *JHBP* tandem clusters. In addition, functional roles of these *BBP* and *JHBP* genes in the cluster were further investigated using *in vivo* electroporation-mediated RNA interference (RNAi) (Ando and Fujiwara 2013). Also, a comparative RNA-seq analysis spanning developmental stages and larval body regions was performed to provide complementary support for the findings obtained from the parallel assay. Taken together, our results demonstrated that although the overall cluster structure of *BBP* and *JHBP* genes is conserved among *Papilio* species, a regulatory divergence in the expression of specific *BBPs* and *JHBPs* underlies the heterochronic diversification of larval green coloration in *Papilio* butterflies.

## Results

### Inhibition of Color Pattern Switch by JH-analog Application

It has been suggested that JH-analog application within the JHSP prevents the color pattern switch from the mimetic pattern (up to 4th instar) to the camouflage pattern (5th instar) in *P*. *xuthus* (Futahashi and Fujiwara 2008). To test whether a rapid decline in JH titer following the 3rd molt triggers a similar pattern switch in *P. memnon*, we applied JH-analog to the larval body at varying intervals after the 3rd molt (Fig. 1b). All larvae that were exposed to JH-analog 24 h after the 3rd molt switched to the normal camouflage pattern (Fig. 1c). However, the application of the JH-analog at 4 and 12 h post-molt inhibited the color pattern switch, retaining the mimetic pattern despite being 5th instar larvae (Fig. 1c and Fig. S1). Notably, two of these five individuals underwent an additional molt to a sixth instar, which is consistent with the established role of juvenile hormone in regulating *Papilio* larval molting (Futahashi and Fujiwara 2008; Fig. S1).

### Genome Annotation in *P*. *memnon*, *P*. *polytes* and *P*. *xuthus*

Previous genome annotations for *P. memnon*, *P. polytes*, and *P. xuthus* remain relatively incomplete and might limit the power to identify genes involved in green coloration. Specifically, the annotation completeness of *P. memnon* from previous research (Iijima et al. 2018) using BUSCO v5.4.3 showed an 88.9% completeness rate, indicating potential for further improvement. Therefore, we reannotated the corresponding *Papilio* genome assemblies to enhance accuracy and facilitate downstream analyses. Re-annotation of the *P. memnon* genome substantially increased the annotated gene number from 12,421 to 12,943, and BUSCO completeness rose to 97.6% (Tables S1 and S2). Similarly, re-annotation increased the annotated gene number from 12,178 to 12,512 in *P. polytes*, and from 12,804 to 12,917 in *P. xuthus* (Table S1).

### Green Color-Related Genes Exploration by RNA-Seq

Next, to explore whether the reported *BBP* and *JHBP* gene families are involved in the green coloration of *P*. *memnon* larvae, we conducted RNA-seq to search for genes consistently expressed in larval regions that display green pigmentation across developmental stages. These regions included the posterior regions of 3rd and 4th instar larvae and the anterior region of 5th instar larvae (Fig. 2a, Table S3). 504 green color-related genes were found to share elevated expression in the green region of all three instars (Table S4). Among these 504 genes, five genes were detected as “*bilin-binding protein-like*,” which are considered homologs of *BBP* and likely involved in blue pigmentation as reported in *P. polytes* (Fig. 2b; Table S4) (Yoda et al. 2020). Additionally, four homologs of *JHBP*s, which are associated with yellow pigmentation in *P. polytes*, were also found in green color-related genes (Fig. 2b; Table S4).

**Fig. 2.**
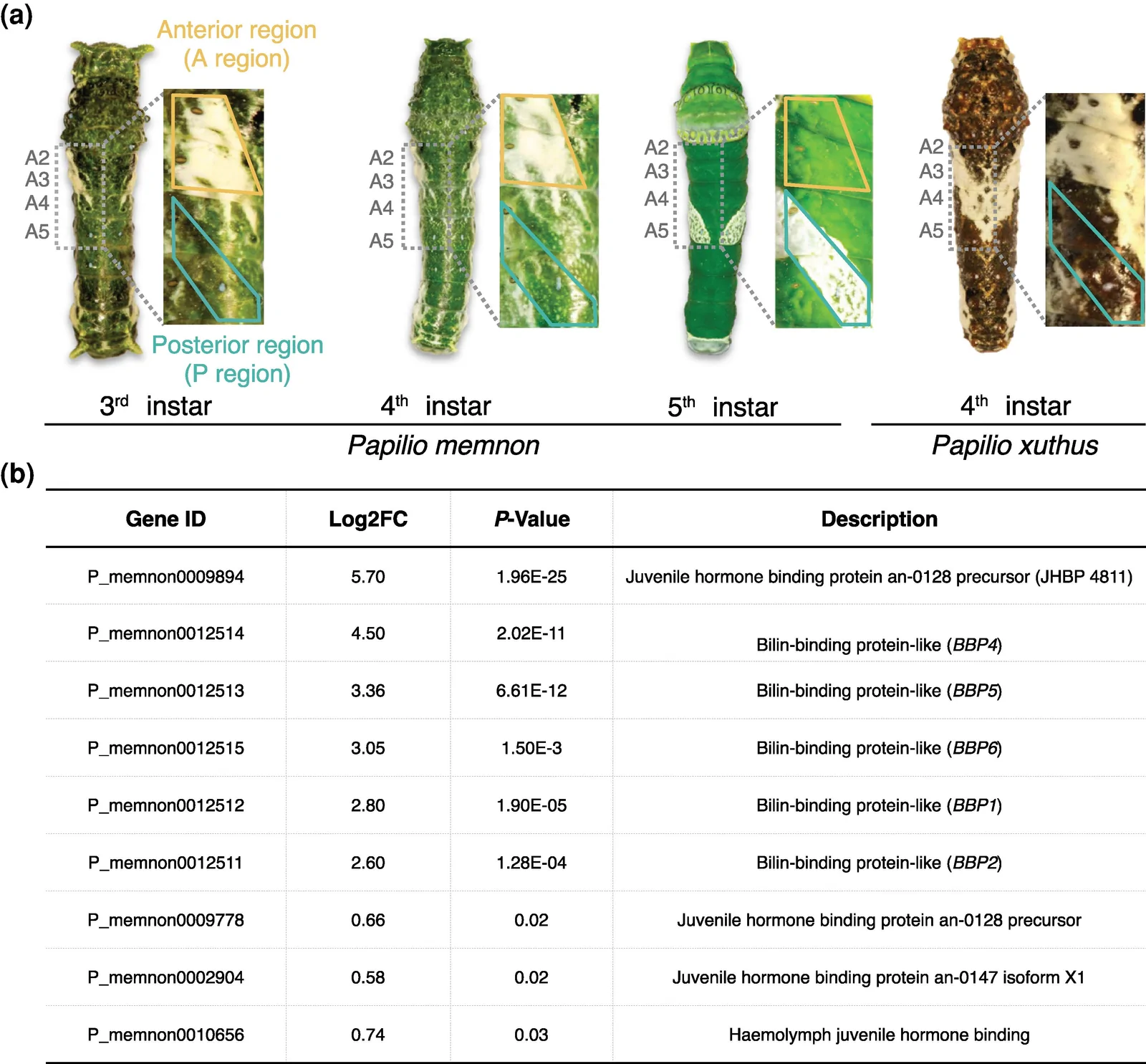
Green color-associated genes. a) Sampling strategy for RNA-seq. Dorsal views of 3rd, 4th and 5th instar larvae of *P. memnon* and 4th instar larvae of *P. xuthus* are shown in the left and right panel, respectively. Boxes represent epidermal regions collected for RNA-seq. The anterior region (A region, across the second and third abdominal segment) is white in the 3rd and 4th instar, but switches to green color in the 5th instar, The posterior region (P region, across the 4th and 5th abdominal segment) is green in the 3rd and 4th instar, but switch to white color in 5th instar. b) *Bilin binding protein* (*BBP*) and *juvenile binding protein* (*JHBP*) genes that were consistently expressed in the green region of 3rd to 5th instar larvae (P region in 3rd and 4th instar larvae and A region in 5th instar larvae) in *P*. *memnon*. A complete list of green color-related genes are provided in Table S4.

### Clusters and Expression Analysis of Genes Associated With Green Color

To explore the evolutionary conservation of *BBP* and *JHBP* genes across *Papilio* species, we examined the genomic organization of these genes in five representative *Papilio* genomes including *P. memnon*, *P. polytes*, *P. xuthus*, *P. machaon*, and *P. bianor*. The genome-wide phylogenetic analysis on all lipocalin family genes revealed that *BBP*s form a monophyletic group, and only six *BBP*s, corresponding to *BBP1* to *BBP6* in *P. polytes* (Yoda et al. 2020), were identified across the examined species (Table S5). Further comparison of the chromosomal arrangement of these six *BBP*s across five *Papilio* species demonstrated both convergent and divergent patterns. In *P. memnon*, *P. polytes*, and *P. xuthus*, the six *BBP*s are arranged tandemly in the same transcriptional orientation and gene order (Fig. 3a). A similar tandem cluster of *BBP*s was also observed in *P. machaon*. However, the gene located between *BBP5* and *BBP6*, although exhibiting sequence similarity to *BBP4* based on alignment, could not be confidently assigned to any *BBP* homolog in the phylogenetic analysis, suggesting potential diversification in its sequence (Fig. 3a; Fig. S2). In contrast, *P. bianor* presented a reduced cluster containing only four tandemly arranged *BBP*s (Fig. 3a; Fig. S2).

**Fig. 3.**
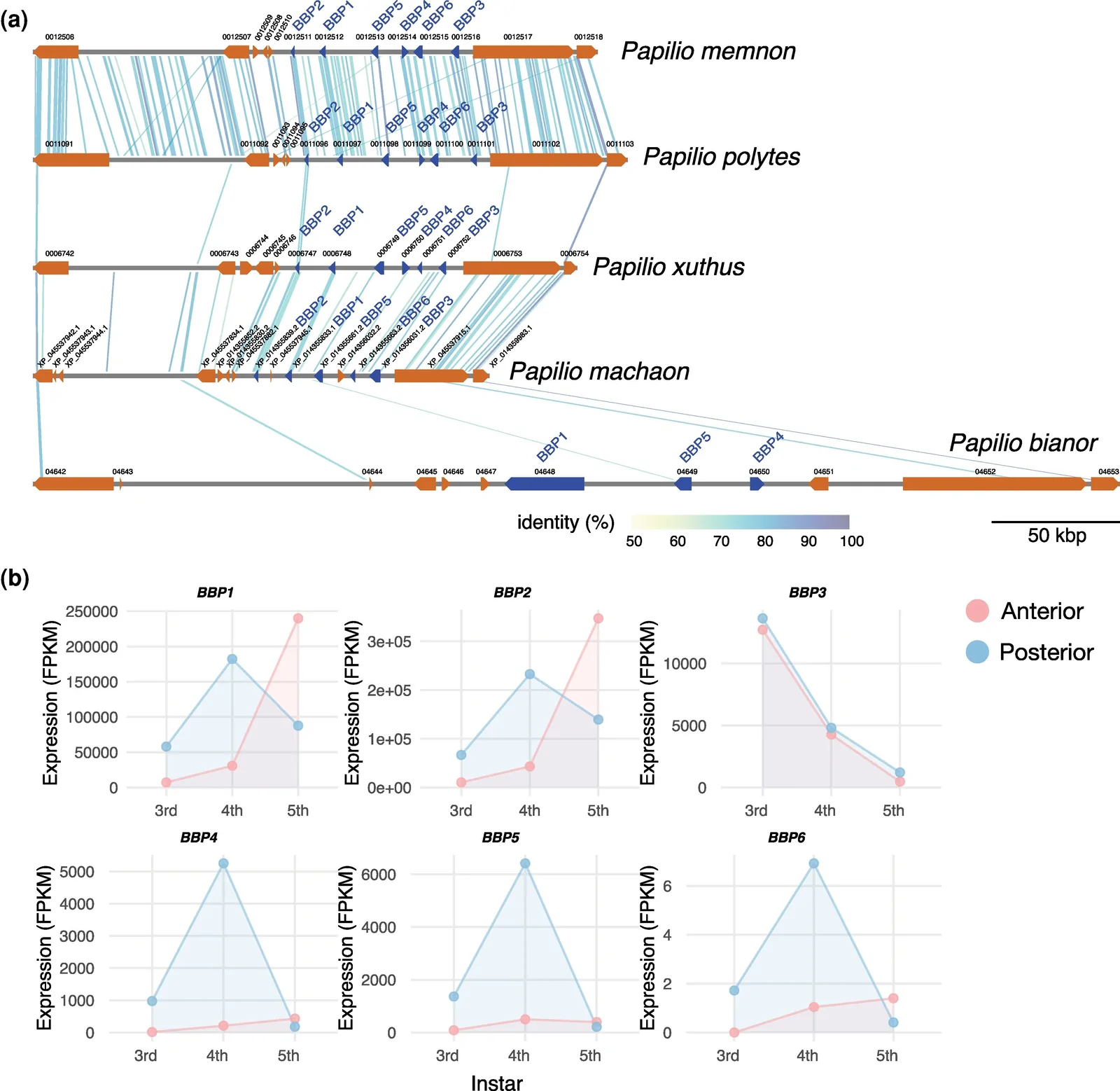
Structure and expression profiles of *BBP*s. a) Genomic organization of *BBP* gene clusters in *Papilio* species. The direction of arrowheads indicates transcriptional orientation. The blastn results showing hits with an identity of 50% or higher and an alignment length of 500 bp or longer are displayed using A-liner (Okuno et al. 2026). b) Expression profiles of *BBP*s in anterior and posterior regions (Fig. 2a) of *P*. *memnon* based on RNA-seq. Expression levels of *BBP*s are shown as the fragments per kilobase of exon per million mapped reads (FPKM).

Similar to the lipocalin gene family, 8 *JHBP* family genes, including *JHBP4811*, *JHBP4812*, *JHBP4813*, *JHBP4814* (*CBP2*), *JHBP4815* (*CBP1*), *JHBP4816*, *JHBP4817*, and *JHBP4818*, also form a monophyletic group, with two to three additional closely related *JHBP*s identified near *JHBP4817* and *JHBP4818* (Table S6). The number of tandemly arranged *JHBP*s varied among species: 8 in *P. memnon*, 10 in *P. polytes*, 9 in *P. xuthus*, 12 in *P. machaon*, and 9 in *P. bianor* (Fig. S4a). Despite these variations, homologs of *CBP1* and *CBP2* were consistently retained within the same cluster in all five species (Fig. S4a).

### Expression Dynamics of Pigmentation-Related Genes in *P. memnon*

To characterize the temporal and spatial regulation of pigmentation-related genes, especially the tandemly arranged *BBP* and *JHBP* genes, we also analyzed their expression dynamics across different larval stages of *P. memnon*. *BBP1* and *BBP2* were found to be prominently expressed in the green regions, specifically, the posterior region of 3rd and 4th instar larvae and the anterior region of 5th instar larvae (Figs. 2a and 3b). Similarly, *BBP4*, *BBP5*, and *BBP6* showed higher expression in the green (posterior) regions than in the white (anterior) regions during the 3rd and 4th instars, but their expression levels declined substantially in both anterior and posterior regions by the 5th instar (Fig. 3b). In contrast, *BBP3* exhibited a relatively uniform expression pattern in anterior and posterior regions with a decrease across developmental stages (Fig. 3b). Furthermore, eight *JHBP*s in the *P. memnon* cluster also demonstrated distinct stage- and region-specific expression patterns. *JHBP4811*, *JHBP4812*, and *JHBP4813* exhibited low expression during the 3rd and 4th instars but were strongly upregulated in the green (anterior) regions during the 5th instar (Fig. S4b). Unlike the other *JHBP*s, *JHBP4815* (*CBP1*) displayed consistently high expression in green regions, with a clear spatial shift from the posterior region in the 4th instar to the anterior region in the 5th instar (Fig. S4b).

In addition to these clustered genes, we examined the expression of lipocalin and *JHBP* gene family members located outside the *BBP* and *JHBP* clusters. Within the lipocalin gene family, *P_memnon0000118* and *P_memnon0012507* were highly expressed in the green regions, specifically the posterior regions during the 3rd and 4th instars and the anterior region during the 5th instar (Fig. 2a and Fig. S5a, c). Among the *JHBP* gene family, none except *JHBP4815* (*CBP1*) showed consistently high expression in the green regions of all developmental stages examined (Fig. S6a, c). However, several other *JHBP* family genes such as *P_memnon0000798*, *P_memnon0002904*, *P_memnon0003392*, *P_memnon0003393*, *P_memnon0003394* and *P_memnon0003395* exhibited higher expression levels in the green regions compared with the white regions within the 3rd and 4th instar (Fig. S6a, c).

Among the melanin-related genes, the expression of *P_memnon0000482* (*laccase2*), *P_memnon0003828* (*TH*), *P_memnon0004843* (*tan*), and *P_memnon0007552* (*dopa decarboxylase*, *DDC*) gradually decreased from the 3rd to the 5th instar in *P. memnon*, coinciding with the fading of black pigmentation and the accumulation of green coloration (Fig. S7a, c). In *P. xuthus*, several melanin-associated genes, including *P_xuthus0001953* (*tan*), *P_xuthus0006002* (*yellow*), *P_xuthus0006005* (*yellow*), and *P_xuthus0011654* (*TH*), exhibited substantially higher expression during the 4th instar compared with other members in the melanin synthesis pathway, potentially suggesting a stronger contribution to the development of black pigmentation (Fig. S7b, d).

### Higher Expression of *BBP*s and *JHBP*s in 4th Instar Larvae of *P. memnon* Than *P. xuthus*

We next compared the expression of *BBP*s in the posterior region of 4th instar larvae, which corresponds to the green region in *P*. *memnon* and black/brown region in *P*. *xuthus* (Fig. 2a, Fig. S5b, d). All six *BBP*s were highly expressed in *P*. *memnon*, whereas their expression was nearly undetectable in *P*. *xuthus* (Fig. 4a). In particular, *BBP1* and *BBP2* showed markedly higher expression in *P*. *memnon*, with expression levels approximately 5- to 6-fold higher than *BBP3*, *BBP4*, *BBP5* and other lipocalin family genes (Fig. 4a and Fig. S5). *BBP6* also demonstrated higher expression in *P*. *memnon* than in *P*. *xuthus*, although its overall expression level is low (Fig. 4a). Furthermore, no other lipocalin family genes outside the *BBP* cluster were found to reach an expression level comparable to *BBP1* and *BBP2* in either *P. memnon* or *P. xuthus* (Fig. S5).

**Fig. 4.**
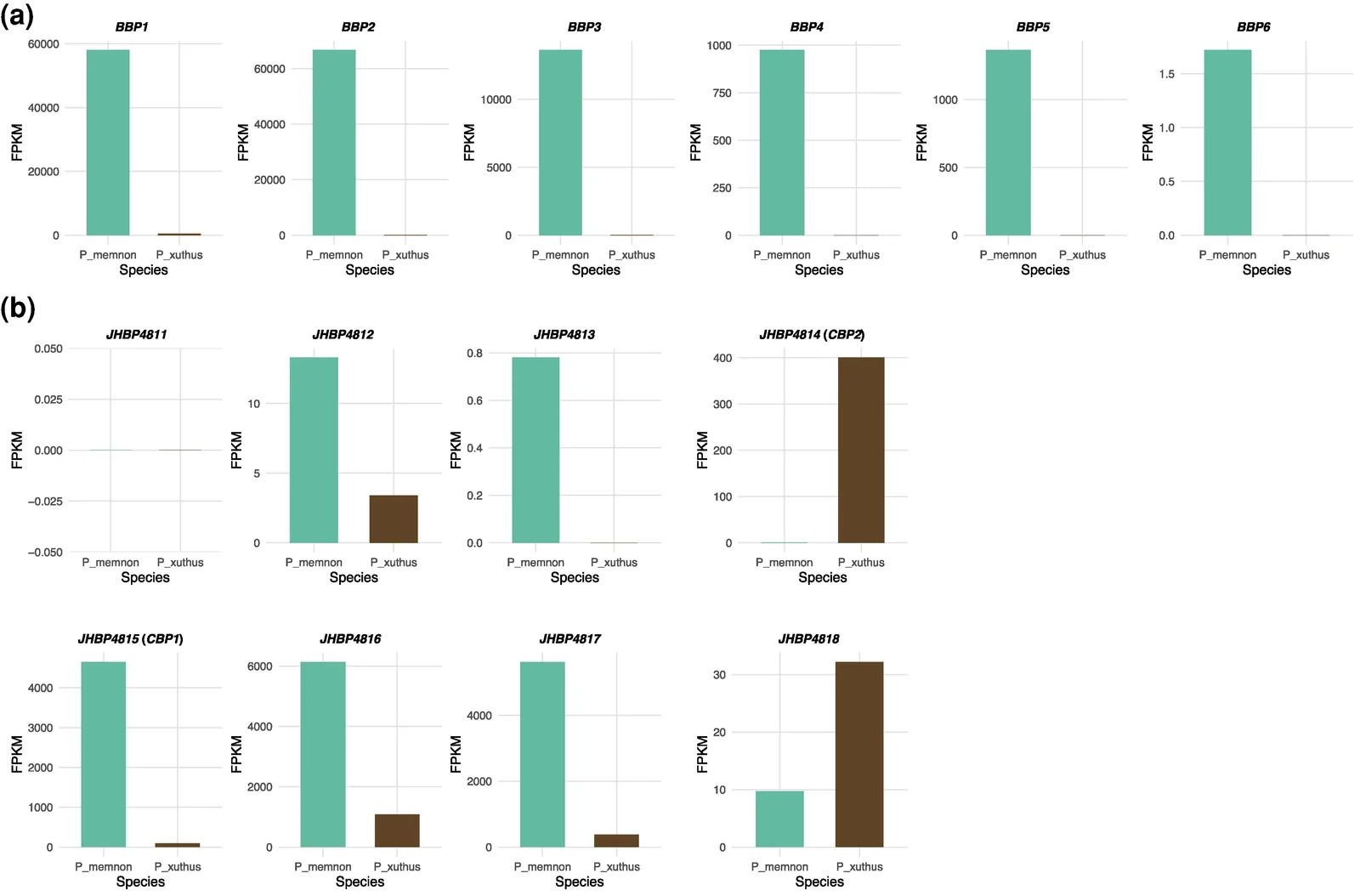
Expression variation of *bilin binding protein*s (*BBP*s) and *juvenile binding protein* (*JHBP*) between *P. memnon* and *P. xuthus*. a) Expression of *BBP*s. All six BBPs showed higher expression in *P. memnon*. b) Expression of *JHBP*s. *JHBP* expression was generally higher in *P. memnon*, except for *JHBP4814* and *JHBP4818*, which were elevated in *P. xuthus*. Expression data are based on RNA-seq in the posterior regions of 4th instar larvae (Fig. 2a) and shown as fragments per kilobase of exon per million mapped reads (FPKM).

Among the eight *JHBP*s within the cluster, *JHBP4812*, *JHBP4813*, *JHBP4815* (*CBP1*), *JHBP4816*, and *JHBP4817* exhibited higher expression in *P. memnon*, while *JHBP4814* (*CBP2*) and *JHBP4818* were more highly expressed in *P. xuthus* (Fig. 4b). Within *P*. *memnon*, *JHBP4815* (*CBP1*), *JHBP4816*, and *JHBP4817* displayed relatively high expression compared with other cluster genes (Fig. 4b). In contrast, although *JHBP4814* (*CBP2*) and *JHBP4818* exhibited higher expression in *P*. *xuthus* than in *P*. *memnon*, their absolute expression levels remained relatively low in the 4th instar, suggesting that they may have a limited role in larval yellow pigmentation. For the other *JHBP* family genes located outside the tandem cluster, the absolute expression levels are consistently low in both species (Fig. S6).

### Functional Roles of *BBP*s and *JHBP*s in Pigmentation

To assess the functional roles of *BBP* and *JHBP* genes in the larval green pigmentation, we performed knockdown using electroporation-mediated RNAi (Ando and Fujiwara 2013). Among the *BBP*s, *BBP1*, and *BBP2*, which showed consistently high expression in the green regions across all stages (3rd, 4th, and 5th instar), were selected for functional analysis first. Independent knockdown of these two genes in 3rd instar larvae resulted in a distinct color shift from green to yellow in the targeting regions (Fig. 5a and b, Figs. S8 and S9), indicating an inhibition of the blue pigmentation. Interestingly, a similar phenotype was observed in both 4th and 5th instar larvae, suggesting that *BBP1* and *BBP2* are critical for maintaining blue pigmentation throughout larval development (Fig. 5a and b, Figs. S8 and S9). In contrast, no clear phenotypic changes were observed for other *BBP*s (Fig. 7). Co-knockdown of *BBP3* and *BBP5* induced only a slight reduction in the blue pigmentation of the green regions, with the change being less pronounced than that of *BBP1* and *BBP2* (Fig. 5c and Fig. S10). Moreover, neither independent nor co-knockdown of *BBP4* and *BBP6* resulted in visible alterations of pigmentation, indicating limited involvement of these genes in green color formation (Fig. S11).

**Fig. 5.**
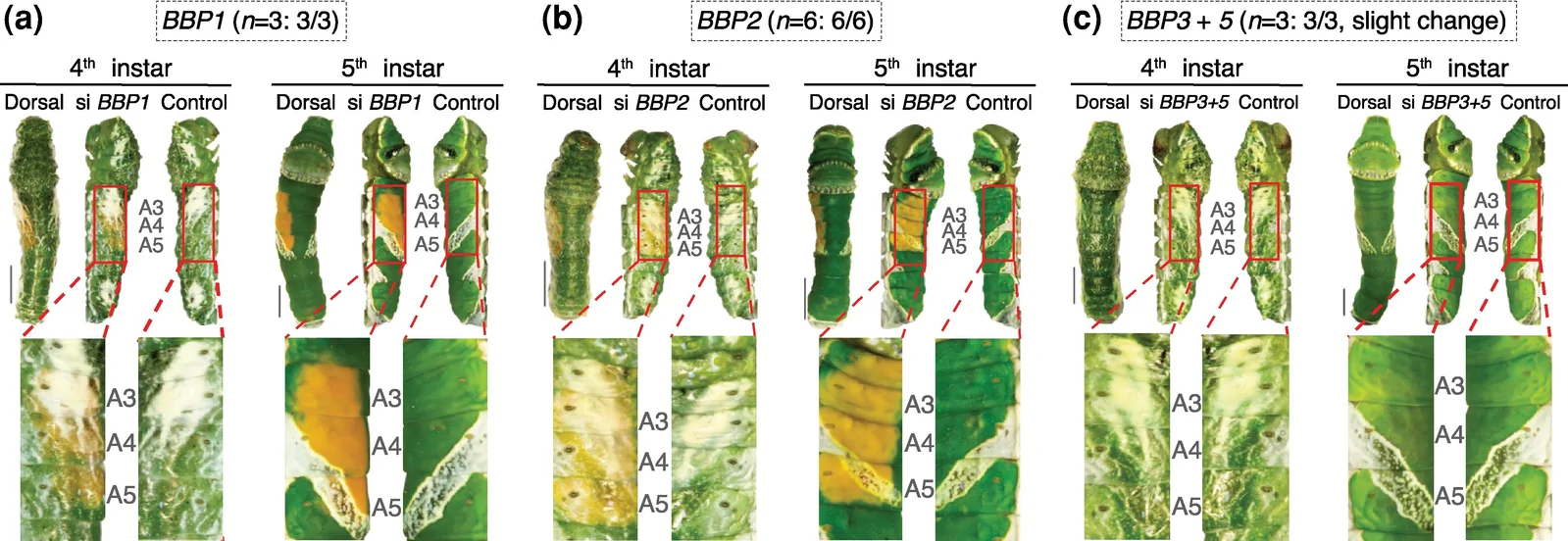
*In vivo* electroporation-mediated RNAi of *BBP*s. a) Phenotypes of 4th instar (left) and 5th instar (right) larvae following RNAi of *BBP1*. b) Phenotypes of 4th instar (left) and 5th instar (right) larvae following RNAi of *BBP2*. c) Phenotypes of 4th instar (left) and 5th instar (right) larvae following multiple-RNAi of *BBP3* and *BBP5*. siRNA was delivered by injection through the intersegmental membrane between the 7th and 8th abdominal segment and introduced into the target epidermal regions via electroporation during the 3rd instar scale bar = 5 mm. Numbers in parentheses indicate the proportion of individuals showing phenotypic changes (e.g. 3/3 for *BBP1* knockdown). Additional biological replicates are shown in Figs. S8–S10.

Among all the *JHBP*s located within the tandem cluster, only knockdown of *JHBP4815* (*CBP1*) led to a shift of larval green regions toward a bluish color for both 4th and 5th instar, suggesting a disruption in yellow pigmentation (Figs. 6a, 7 and Fig. S12). In contrast, no clear phenotypic changes were observed in larvae following knockdowns of the other *JHBP*s (Figs. 6b, 7 and Fig. S13). However, knockdowns of either *JHBP4811* or *JHBP4813* caused pupal green coloration to turn slightly blue, indicating the impaired yellow pigment at pupal stages (Fig. 7 and Fig. S14). For all knockdown experiments, we confirmed a significant (Student's *t*-test, *P*-value < 0.05) reduced expression of the target genes within the treated regions by qPCR (Fig. S15). Individuals treated with universal siRNA exhibited no visible changes in epidermal pigmentation, indicating that electroporation alone did not induce phenotypic effects (Fig. S16).

**Fig. 6.**
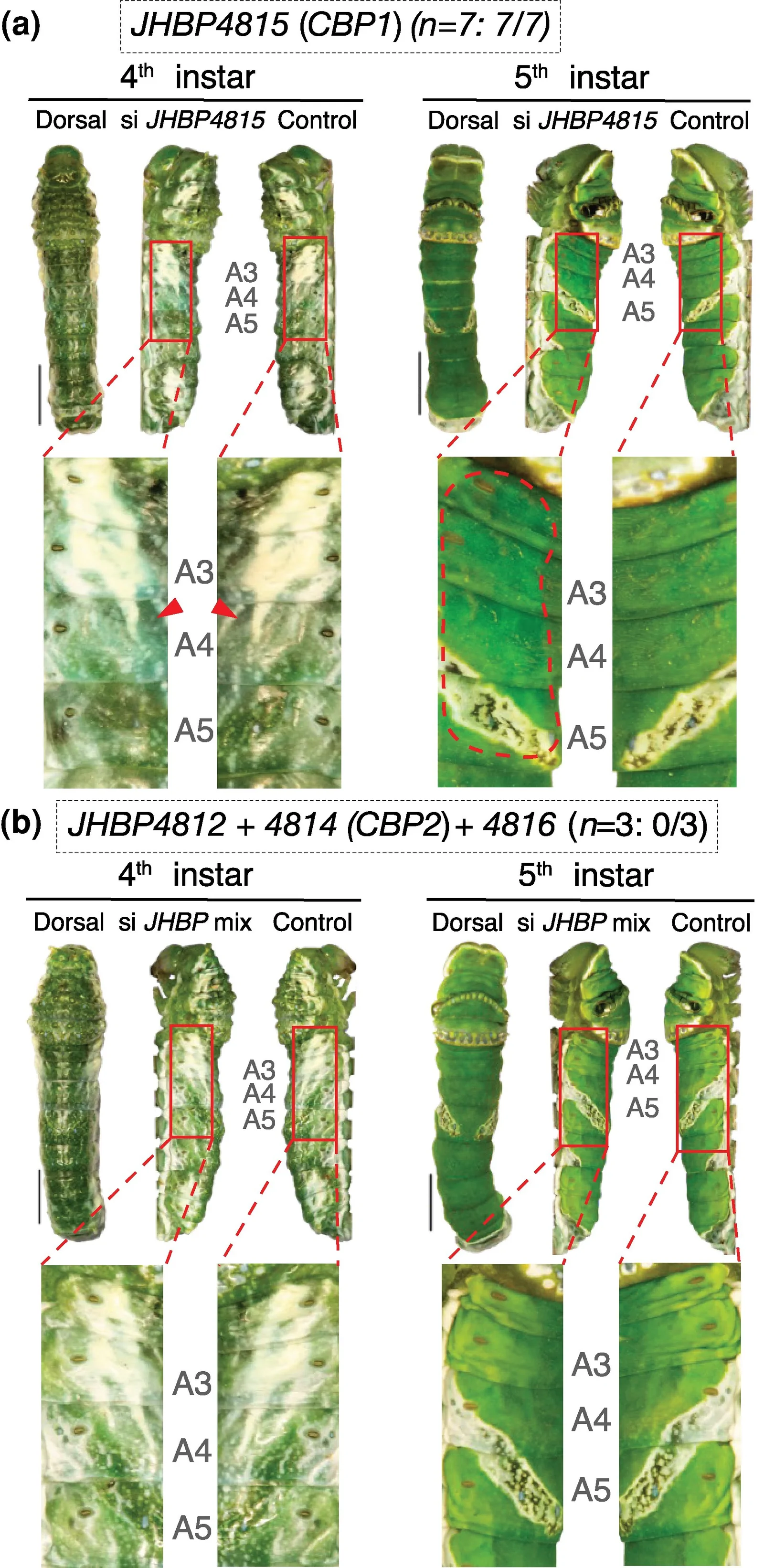
*In vivo* electroporation-mediated RNAi of *JHBP*s. a) Phenotypes of 4th instar (left) and 5th instar (right) larvae following RNAi of *JHBP4815*. b) Phenotypes of 4th instar (left) and 5th instar (right) larvae following multiple-RNAi of *JHBP4812*, *JHBP4814* and *JHBP4816*. siRNA was delivered by injection through the intersegmental membrane between the 7th and 8th abdominal segment and introduced into specific the target epidermal regions via electroporation during the 3rd instar, scale bar = 5 mm. Among all the *JHBP*-targeted RNAi experiments, only the knockdown of *JHBP4815* caused a slight reduction of yellow color during the larval stage. Numbers in parentheses indicate the proportion of individuals showing phenotypic changes (e.g. 7/7 for *JHBP4815* knockdown). Additional biological replicates are shown in Fig. S12 and S13.

**Fig. 7.**
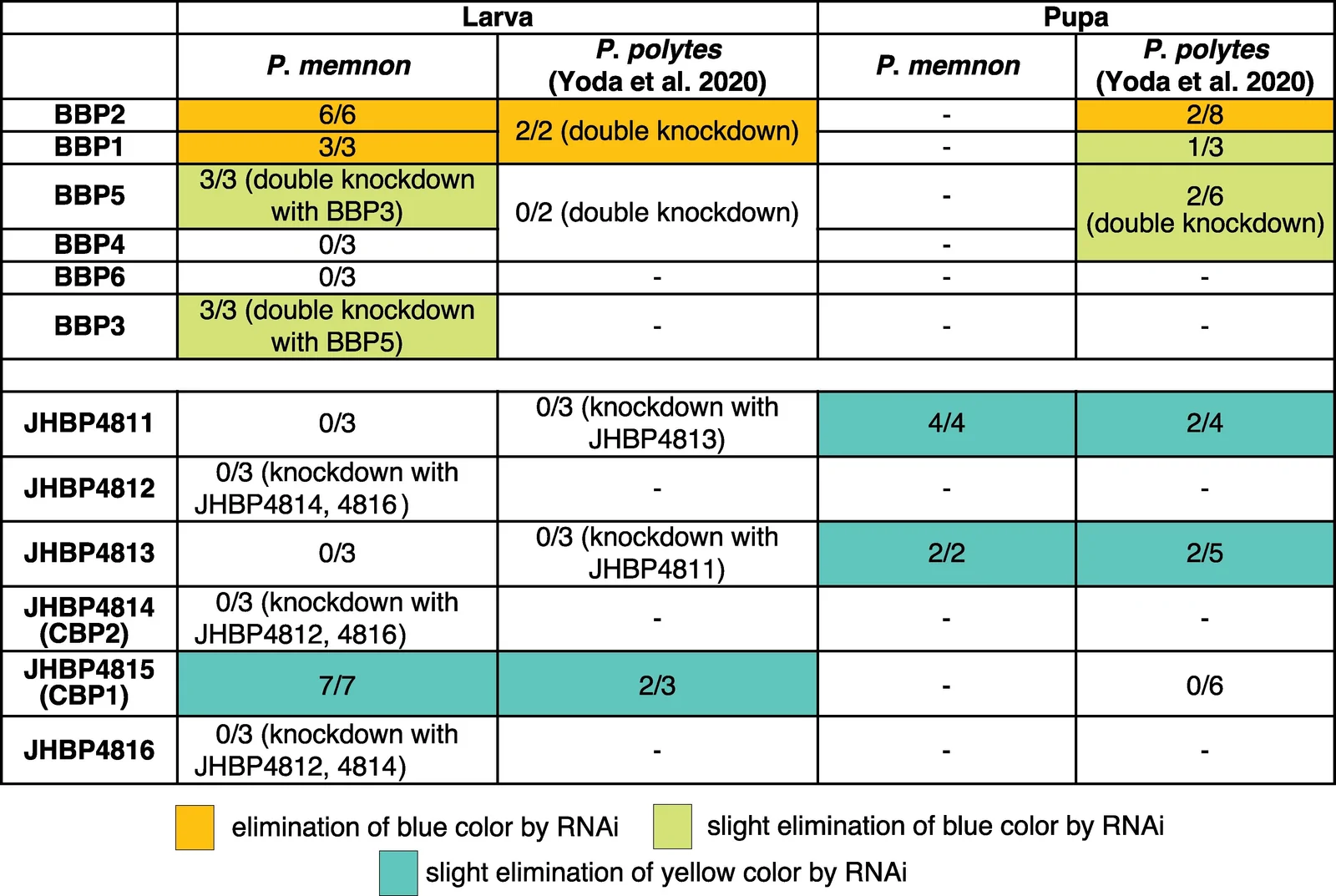
Summary of the functional roles of *BBP*s and *JHBP*s in *P*. *memnon* and *P*. *polytes* based on this study and Yoda et al., (2020). Knockdown of *BBP*1 and *BBP*2 caused pronounced phenotypic changes in both species, confirming their essential roles in larval green coloration. *BBP5* knockdown caused a mild effect in *P. memnon* but no detectable effect in *P. polytes*. *JHBP4815* knockdown affected yellow pigmentation in larvae but not in the pupae.

## Discussion

The evolution of caterpillar coloration has long been implicated in predator avoidance, with mimicry and camouflage playing central roles in larval adaptive strategies (Caro and Koneru 2021; Robinson et al. 2023). In this study, we provided direct evidence that green pigmentation in *Papilio* larvae is regulated by the coordinated expression of multiple *BBP* and *JHBP* genes, with notable species-specific differences in their expression timing and function (Fig. 7). These findings highlight that regulatory divergence, rather than changes in gene content, can also drive the evolution of ecologically important traits in closely related species (Shirataki et al. 2010; Zheng et al. 2011; Liu et al. 2026).

The rapid drop of JH titer at the beginning of the 4th instar was demonstrated as a critical regulatory event responsible for the switch from black/brown to green camouflage patterns in *P. xuthus* (Futahashi and Fujiwara 2008; Jin et al. 2019). This hormonal transition has also been hypothesized to mediate the expression of downstream pigmentation genes essential for final-instar pattern formation (Jin et al. 2019). Although *P. memnon* larvae show an earlier accumulation of green coloration, our JH analog application experiments suggested that a similar reduction in JH titer occurring approximately 12 h after the 3rd molt triggers the transition from the mimetic-like to the camouflage phenotype. Our results indicate that the transcriptional activation of *BBP* and *JHBP* genes is not solely governed by JH signaling in *P. memnon*, although the direct regulatory effects of JH on *BBP* and *JHBP* expression remain to be investigated. It may suggest the involvement of additional regulatory factors that facilitate this precocious development of green pigmentation. In *P. polytes*, for instance, the pupal brown/green color switch is known to involve pupal cuticle melanizing hormone (PCMH) (Awiti and Hidaka 1982; Starnecker and Hazel 1999; Yamanaka et al. 1999). Further investigation into the upstream regulators and signaling cascades underlying this heterochronic shift will be essential to understand the evolution of adaptive green coloration in *Papilio* larvae.

In the common ancestor of *Papilio* butterflies, green pigmentation genes essential for larval and pupal camouflage likely diverged and became organized into tandem clusters. Despite the temporal expression dynamics, the genomic organization of the *BBP* and *JHBP* clusters, including gene number, order, or orientation, is conserved across all three species, *P. memnon*, *P. xuthus*, and *P. polytes* (Fig. 3a and Fig. S4). This conserved pattern suggests that regulatory changes, rather than structural differences in gene content, are responsible for the heterochronic shift in pigmentation of *P. memnon*. Moreover, most *BBP*s within the cluster exhibited dynamic but consistent expression patterns across larval development, with transcript levels peaking during the 4th instar (Fig. 3b), suggesting a shared regulatory factor may exist to co-regulate coordinated expression patterns. One possibility is that these *BBP* genes within the cluster contain common upstream regulatory motifs responsive to an early-acting transcription factor (Marco et al. 2009; Liu et al. 2025), whose earlier activation in *P. memnon* may initiate cluster-wide transcription and, consequently, lead to earlier green pigment accumulation. Alternatively, given that the *BBP* and *JHBP* gene families are induced while the brownish pattern is concurrently suppressed, the regulation may involve a more broadly acting factor (e.g. a hormone) that controls pigment pathway transitions at the systemic level.

Unlike *BBP*s, *JHBP*s may function in a stage-specific manner, with distinct cluster genes involved in yellow pigmentation during the larval and pupal stages (Fig. 7). In both *P*. *memnon* and *P*. *polytes*, *JHBP4815* (*CBP1*) is involved in yellow pigment synthesis during the larval stage, while *JHBP4811* and *JHBP4812* act during the pupal stage (Figs. 6 and 7). The differential roles of *JHBP4815* in larvae and *JHBP4811* and *JHBP4813* in pupae indicate an evolutionary diversification in which *JHBP* paralogs acquired distinct stage-specific functions. Moreover, the knockdown of *JHBP4815* did not completely eliminate the yellow pigmentation, in contrast to the striking color shift observed with *BBP1* and *BBP2* knockdown (Fig. 6a). This partial phenotypic change suggests that other, as yet uncharacterized, *JHBP*s may also contribute to larval yellow pigmentation. Additional functional assays on other *JHBP* family members with green-specific expression (Fig. S7) together with direct pigment quantification and protein–pigment binding assays, will help fully clarify their functional roles.

In addition to the diversification, functional redundancy of multigene families may also contribute to adaptive evolution by buffering against deleterious mutations and providing regulatory flexibility under selective pressure (Nowak et al. 1997; Zhou et al. 2012; Láruson et al. 2020). The *BBP* and *JHBP* gene clusters examined in this study likely arose through tandem duplication events. Our functional analysis demonstrated that only *BBP1* and *BBP2* produced pronounced phenotypic effects upon knockdown, leading to a clear reduction in blue pigmentation in *P. memnon* larvae (Fig. 7). These results are consistent with the transcriptomic data showing that *BBP1* and *BBP2* exhibited the highest expression levels within the whole *BBP* cluster (Fig. 3b). Moreover, *BBP1* and *BBP2* are also involved in the pupal blue coloration in *P. polytes* (Yoda et al. 2020), which aligned with the observation that only *BBP1* and *BBP2* retained the high expression in the final (5th) instar (Fig. 3b). In contrast, independent knockdowns of *BBP3* and *BBP5* had no detectable phenotypic effects, while co-knockdown of these two genes resulted in a subtle reduction of blue coloration (Fig. 5c), indicating functional redundancy. Collectively, these results support the conclusion that *BBP1* and *BBP2* are the primary regulators of blue pigmentation in both larval and pupal stages, while additional *BBP*s in the tandem cluster may act redundantly to provide extra evolutionary flexibility.

*P. xuthus* (black/brown) and *P. memnon* (green) exhibit distinct larval body coloration, with differences most pronounced at the 4th instar (Fig. 1a). We found that all *BBP* genes showed substantially higher expression in *P. memnon* compared with *P. xuthus* (Fig. 4a), which, though requiring confirmation with additional biological replicates, supports the potential co-regulation and functional redundancy of *BBP* genes. In contrast, not all *JHBP* family genes showed a similar pattern of elevated expression in *P. memnon* (Fig. 4b). For example, 5 *JHBP*s including *JHBP4815* showed higher expression in *P. memnon*, whereas *JHBP4814* and *JHBP4818* were more strongly expressed in *P. xuthus* (Fig. 4b), suggesting these two genes may have limited roles involved in the yellow pigmentation. The RNAi of *JHBP4814* confirms its limited involvement in yellow pigmentation (Fig. S13). The differential expression of *BBP*s and *JHBP*s between species likely contributes to the earlier onset of green pigmentation observed in the 3rd and 4th instar larvae of *P. memnon*. Moreover, the consistently elevated expression of *BBP* genes together with most *JHBP* genes in *P. memnon* further supports the existence of a shared upstream regulator. Together, these findings suggest that divergence in the temporal activation of a shared upstream regulator that modulates the *BBP* and *JHBP* cluster expression may have driven the adaptive heterochronic shift in green coloration observed in *P. memnon*, thereby contributing to the evolutionary divergence of larval pigmentation between these closely related species. Subtle changes in developmental timing can lead to pronounced phenotypic diversification, ultimately contributing to the generation of biodiversity in nature (Gould 1977; Dobreva et al. 2022; Harry and Zakas 2024). Heterochronic shifts in gene expression have been widely documented as a key driver of both morphological innovation and species divergence across taxa (Wray et al. 2003; Fay and Wittkopp 2008; López-Maury et al. 2008; Hamann et al. 2021). For example, heterochrony has been implicated in the evolution of cranial morphology in diverse species, including fish, birds, mammals, and reptiles, where changes in the timing of developmental programs alter growth trajectories and final anatomical structures (Lieberman et al. 2007; Bhullar et al. 2012; Koyabu et al. 2014; Jékely et al. 2015; Morris et al. 2019). Such developmental shifts can be driven by changes in the temporal regulation of genetic programs, including transcriptional networks and hormone-mediated signaling pathways. This may suggest a recurring evolutionary mode: modifications in the timing of gene expression and hormonal regulation can serve as a flexible mechanism for phenotypic innovation. In this context, our results suggest that the heterochronic shift observed in the BBP/JHBP gene clusters represents a similar mechanism, where changes in the timing of gene expression, potentially mediated by an upstream regulator, contribute to the emergence of the green pigmentation pattern. This supports a broader view in which heterochrony in regulatory networks, rather than changes in gene content alone, plays a central role in shaping phenotypic diversity.

## Materials and Methods

### Butterfly Rearing

*P. memnon* females were collected on Amami-Ohshima Island, Kagoshima, Japan in August 2020. We bred the mother butterflies to lay eggs on fresh citrus leaves in rectangular plastic containers with a size of 30 × 18 × 18 cm^3^, at 25 °C, and under a 16:8 light: dark cycle. The eggs were collected in a round plastic cup (diameter, 100 mm; height, 45 mm) and the hatched caterpillars were fed on fresh citrus leaves until pupation and adult emergence in the rectangular plastic containers at 25 °C and under a 16:8 light: dark cycle.

### Juvenile Hormone (JH) Analog (Fenoxycarb) Treatment

JH analog application experiments were conducted following the previous protocol (Futahashi and Fujiwara 2008) to ascertain the function of JH in switching larval patterns from 4th to 5th instar larvae in *P*. *memnon*. Fenoxycarb (Cayman Chemical Company, Michigan, USA), which is a carbamate insect growth regulator mimicking juvenile hormone (JH) (El-Sheikh et al. 2016), was dissolved in acetone (Wako Pure Chemical Industries, Japan). Then 5 μL of 1μg/μL fenoxycarb was ectopically applied to the larval body at the first 4, 12, and 24 h during the penultimate (4th) instar. Larvae in the control group were treated with 5 μL of acetone only. Larval developmental stage was confirmed by a camera-recording system with a 5-min capture interval (Cosmosoft Technologies, India). JH analog-treated individuals were starved for 24 h and then reared in an independent incubator at 25 °C for further phenotypic observation.

### Tissue Sampling

Epidermal samples were collected from the anterior and posterior epidermal regions of 3rd, 4th, and 5th instar larvae (Fig. 2a), 12 h post-molt to ensure stage-specific consistency (Fig. 1b). Before dissection, all larvae were anesthetized by a 5-minute incubation on ice. The caterpillars were then soaked in the precooled phosphate-buffered saline (PBS) and fixed on the wax dish with insect pins. Under a stereo microscope (Stemi 35, ZEISS, Germany), internal tissues including gut, fat body, and muscles were carefully removed to expose the epidermis without physical damage. The total RNA was extracted using TRI reagent (Sigma-Aldrich, Massachusetts, USA) and treated with DNase I (Takara, Shiga, Japan) following a previously described protocol (Jin et al. 2019).

### Library Preparation for RNA Sequencing (RNA-Seq)

RNA-seq libraries were prepared using the SMART-Seq v4 Ultra Low Input RNA Kit (Takara, Shiga, Japan) and the TruSeq RNA Sample Prep Kit v2 (Illumina, San Diego, CA, USA), according to the manufacturers' protocols. Sequencing was performed by Macrogen Japan on NovaSeq 6000 (Illumina, San Diego, CA, USA) platform with the configuration for 101 bp paired-end reads. Due to the limited RNA yield from epidermal tissue, samples from three individuals were pooled per condition to ensure sufficient sequencing depth and to mitigate the impact of any single individual or technical outlier on the expression profiles (Table S3). Consequently, we were unable to assess inter-individual biological variation and had no statistical power to detect differential gene expression within each developmental stage. The resulting expression profiles were therefore used to characterize condition-level expression patterns rather than for statistical testing of differential expression. The libraries showed increased variability at low expression levels and reduced dispersion at higher expression levels (Fig. S17).

### Genome Annotation

For *P*. *machaon*, the National Center for Biotechnology Information (NCBI) Refseq annotation (NCBI accession: GCF_912999745.1) was used, while the annotation by Lu et al. (2019) was used for *P*. *bianor* (Lu et al. 2019). Gene reannotation was performed in *P*. *memnon*, *P*. *polytes* and *P*. *xuthus* using the GINGER pipeline (Taniguchi et al. 2023). Homology-based predictions were performed using the amino acid sequences in the following species: *Papilio polytes*, *Bombyx mori*, *Papilio memnon*, *Acyrthosiphon pisum*, *Papilio xuthus*, *Apis mellifera*, *Papilio machaon*, *Drosophila melanogaster*, *Papilio bianor*, *Tribolium castaneum*, *Heliconius melpomene*, *Danaus Plexippus*, all retrieved from the NCBI database. The reference genome constructed using Platanus-allee v. 2.3.2 (Kajitani et al. 2019) was used in *P*. *memenon* and *P*. *polytes* as previously described (Komata et al. 2022). NCBI RefSeq assembly was used in *P*. *xuthus* (NCBI accession: GCF_000836235.2). The statistics of the genome annotation are shown in Table S1. Next, the completeness of the annotated genes was evaluated using BUSCO v5.4.3 in the protein mode (Manni et al. 2021) with the lepidoptera_odb10 database (Table S2). Finally, functional annotation was performed by a *blastp* search against KAIKObase (https://sgp.dna.affrc.go.jp/KAIKO/) and gene names were assigned accordingly.

### Transcripts Quantification

Quality control of raw sequencing data was conducted using FastQC v0.11.9 (Andrews 2010). The reads were then mapped to the *P*. *memnon* reference genome (see details above) using STAR v2.7.10b (Dobin et al. 2013), and the abundance of genes was quantified using RSEM (v1.3.1) (Li and Dewey 2011). In this study, there was only one sample per region without replication (although, as described above, each sample consisted of a pool of three individuals). To identify genes associated with green pigmentation, we inferred genes showing higher expression in green regions (posterior region in 3rd and 4th instars and anterior region in 5th instar) across developmental stages using a generalized linear model implemented in edgeR v3.14 (Robinson et al. 2010), with developmental stages included as a covariate. As a result, 504 genes with unadjusted *P*-values < 0.05 were defined as green color-related genes (Table S4). To enable inter-species comparison, gene expression in the black regions of 4th instar larvae of *P. xuthus* was quantified using the same analytical pipeline applied to *P. memnon*. The raw RNA-seq data were from Jin et al. (2019) and mapped to the *P. xuthus* reference genome (NCBI accession: GCF_000836235.2).

### Identification of *JHBP* and *BBP* Cluster

Previous studies suggested that *BBP* and *JHBP* were organized in tandem gene clusters in *P. polytes* (Yoda et al. 2020). To explore the genomic organization of these gene families across *Papilio* species, we identified *BBP* and *JHBP* family genes genome-wide from *P. memnon*, *P. polytes*, *P. xuthus*, *P. machaon*, and *P. bianor* with the improved genome annotation (see details above). In brief, protein sequences from each species were annotated using InterProScan v5.62-94 (Jones et al. 2014). Genes that were classified as members of “lipocalin” and “juvenile hormone binding protein (JHBP)” were selected for further analysis. Multiple sequence alignments for “lipocalin” and “JHBP” genes were created using MAFFT v7.505 (Katoh and Standley 2013) and ambiguous alignment regions were removed using trimAl v1.4. rev15 (Capella-Gutiérrez et al. 2009). Phylogenetic relationships of these genes were inferred via maximum likelihood with IQ-TREE v2.2.5 (Minh et al. 2020).

Within the lipocalin family, *BBP*s were identified based on sequence similarity searches using *blastp* against the KAIKObase database (https://kaikobase.dna.affrc.go.jp/). Their genomic positions were subsequently examined to assess distribution and potential clustering patterns across species. For the identification of the JHBP cluster, we first performed *blastp* searches using the previously characterized carotenoid-binding proteins *CBP1* and *CBP2* from *P. xuthus* (Futahashi et al. 2012) as queries against the *JHBP* family genes identified in this study. The homologs corresponding to *CBP1* and *CBP2* were then used as genomic anchors to locate and define the *JHBP* cluster in each species. To maintain consistency with published annotations, clusters of *BBP*s and *JHBP*s (including *CBP*s), are described using the gene identifiers reported in a previous study (Yoda et al. 2020).

### *In Vivo* Electroporation-Mediated RNA Interference

Target-specific short interfering RNA (siRNA) was designed using the web server siDirect 2.0 (http://sidirect2.rnai.jp/). Design parameters followed established guidelines to enhance efficacy and minimize off-target effects. Specifically, the G/C content of target sequence was subject to 35%–55%. Sequences containing four or more consecutive guanines or cytosines were excluded to facilitate chemical synthesis. A maximum Tm value in seed sequence was set to 21.5 °C to minimize the seed-dependent off-target effects (Ui-Tei et al. 2008). All siRNAs were synthesized through Custom oligonucleotide synthesis service at the Food Assessment & Management Center (FASMAC, Atsugi, Japan). The lyophilized siRNAs were dissolved in the annealing buffer [100 mM CH_3_COOK, 2 mM Mg (CH_3_COO)_2_, 30 mM HEPES-KOH, pH 7.4] to a concentration of 500 μM and then stored at −80 °C until use. Full sequence information of all siRNAs used in this study is listed in Table S7.

Functional analysis of candidate genes was performed using in vivo electroporation-mediated RNAi (Ando and Fujiwara 2013). Injection capillaries were prepared from filament-containing glass capillaries (Narishige Group, Tokyo, Japan) with a versatile puller (Narishige Group, Tokyo, Japan). siRNA was then loaded into the injection capillary and injected with the FemtoJet microinjector (Eppendorf, Hamburg, Germany). *Papilio* larvae were gently fixed on an operational platform with tape. The position of the capillary was controlled by a three-dimensional manual micromanipulator (Narishige Group, Tokyo, Japan). 1 μL of 250 μM siRNA was then injected into the hemolymph of caterpillars through the intersegmental membrane between the 7th and 8th abdominal segments. To minimize tissue damage, two phosphate-buffered saline (PBS) droplets were placed between the electrodes and the larval cuticle, serving as conductive buffers. Electroporation was then performed using a super electroporator (Nepa Gene, Tokyo, Japan), with a poring pulse followed by a transfer pulse to facilitate siRNA uptake by target epidermal cells. Specific electroporation parameters are provided in Table S8. A universal siRNA (Nippon Gene, Tokyo, Japan) was used as a negative control to account for potential nonspecific effects of electroporation (Fig. S16).

### Validation of Knockdown Efficiency by Quantitative Real-Time PCR (RT-qPCR)

The RNA was extracted from the control (nonelectroporated) and electroporated regions of the same individual. Complementary DNA (cDNA) libraries were constructed by following the protocol of Verso cDNA synthesis Kit (Thermo Fisher Scientific, USA) with random hexamers as primers. qPCR reactions were prepared from cDNA templates using Power SYBR Green PCR Master Mix (Thermo Fisher Scientific, USA) according to the manufacturer's protocol and run on the StepOne Real-Time PCR System (Applied Biosystems, Massachusetts, USA) and the QuantStudio 3 Real-Time PCR System (Thermo Fisher Scientific, USA). Briefly, amplification was initiated with a denaturation step at 95 °C for 10 min, followed by 40 cycles of 95 °C for 15 s and 60 °C for 60 s for annealing and extension. Amplification specificity was confirmed via melt curve analysis under the following cycling conditions: 95 °C for 15 s, 60 °C for 1 min, and 95 °C for 15 s. Raw data were processed using StepOne Software v2.1 and QuantStudio Design and Analysis Software v1.1, corresponding to each qPCR system, respectively. Relative expression levels of target genes were quantified using a standard curve generated from serially diluted cDNA samples (1×, 4×, 16×, 64×, and 256×). The *ribosomal protein L3* (*rpL3*) gene was selected as an endogenous reference, based on its stable expression across multiple cell types (Thorrez et al. 2008). Primers used for qPCR are listed in Table S9.

## Supplementary Material

evag253_Supplementary_Data

## Data Availability

The raw RNA-seq data generated for this study have been deposited in the NCBI repositories (NCBI accession: PRJNA964473). All RNA-seq data of *P. xuthus* are available under the NCBI accession PRJNA1428501. The genome assemblies and annotation files used in this study are available from Zenodo at https://doi.org/10.5281/zenodo.22821982 (Komata 2026).
