# Supplementary material for: Molecular Mechanisms Driving Larval Green Color Adaptation in Swallowtail Butterfly, Papilio memnon
Source: Genome Biol Evol. 2026 Oct 13;18(10):evag253. doi: 10.1093/gbe/evag253 (PMC13717194; doi:10.1093/gbe/evag253)
Supplement: Supplementary file 1 [file evag253_supplementary_data.zip › 260714_pme_SI.pdf]

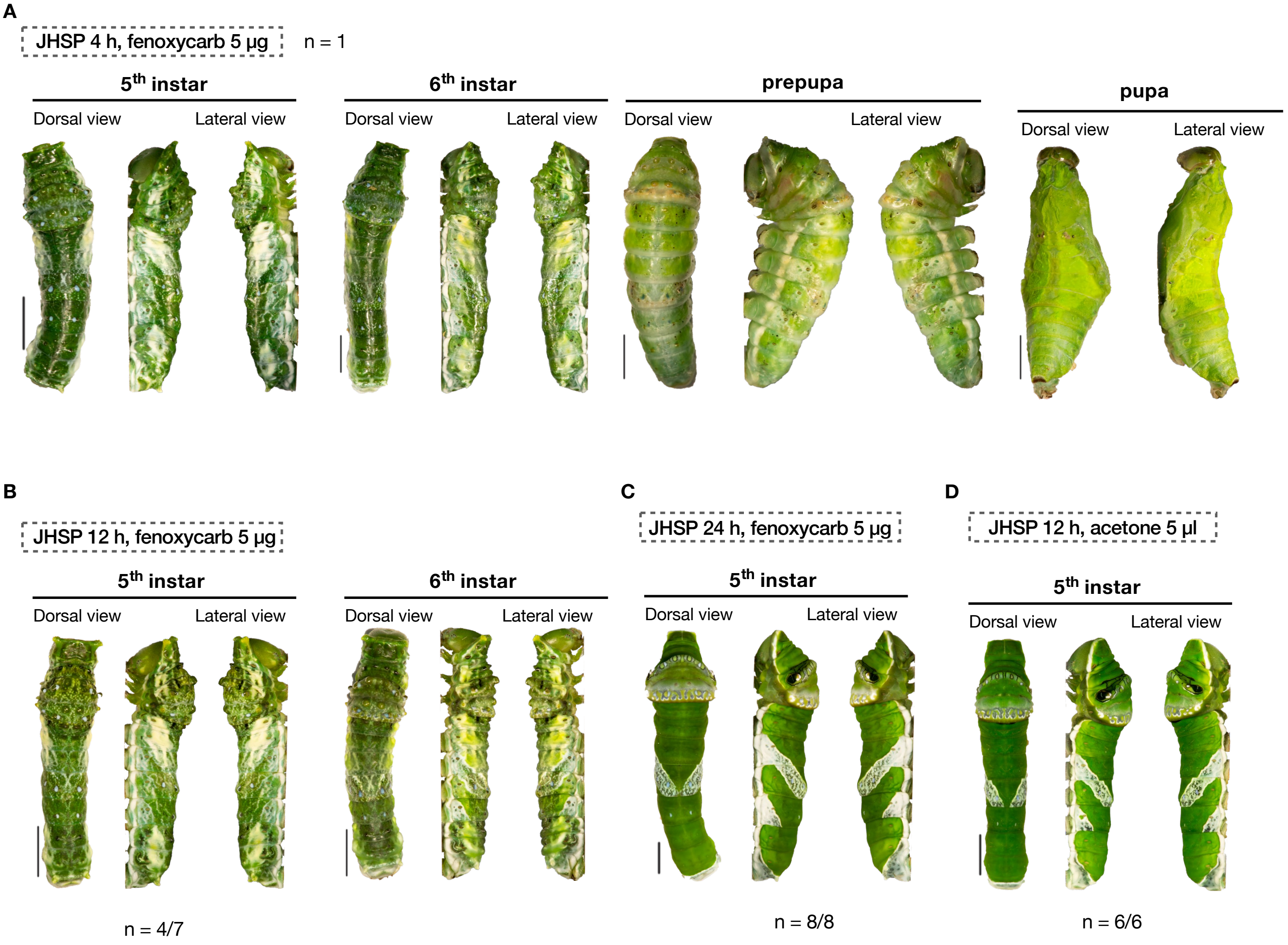

Figure S1. Juvenile Hormone-analog (JHA) treatment in *P. memnon*. (A) Individuals treated with 5 µg fenoxycarb at 4 h after the 3rd molt (n = 5). Five individuals were treated at 4 h after the 3rd molt, but four died. The sole surviving individual became a 6th instar larva, which is not typically seen, and pupated without becoming camouflaged that is the normal color pattern of five instar larvae. (B) Individuals treated with 5 µg fenoxycarb at 12 h after the 3rd molt. Switching to the camouflage pattern did not occur in four of the seven individuals, and the 5th instar larvae had the same color pattern as the 4th instar larvae. Of the four individuals for which switching did not occur, one died after reaching 6th instar larvae and the other three died at 5th instar. (C) Individuals treated with 5 µg fenoxycarb at 24 h after the 3rd molt. (D) Individuals treated with 5 µg acetone 12 h after the 3rd molt. JHSP: Juvenile hormone sensitive period. The color pattern switch (from mimetic to camouflage) was inhibited by the JHA treatment during JHSP, generating an additional instar.

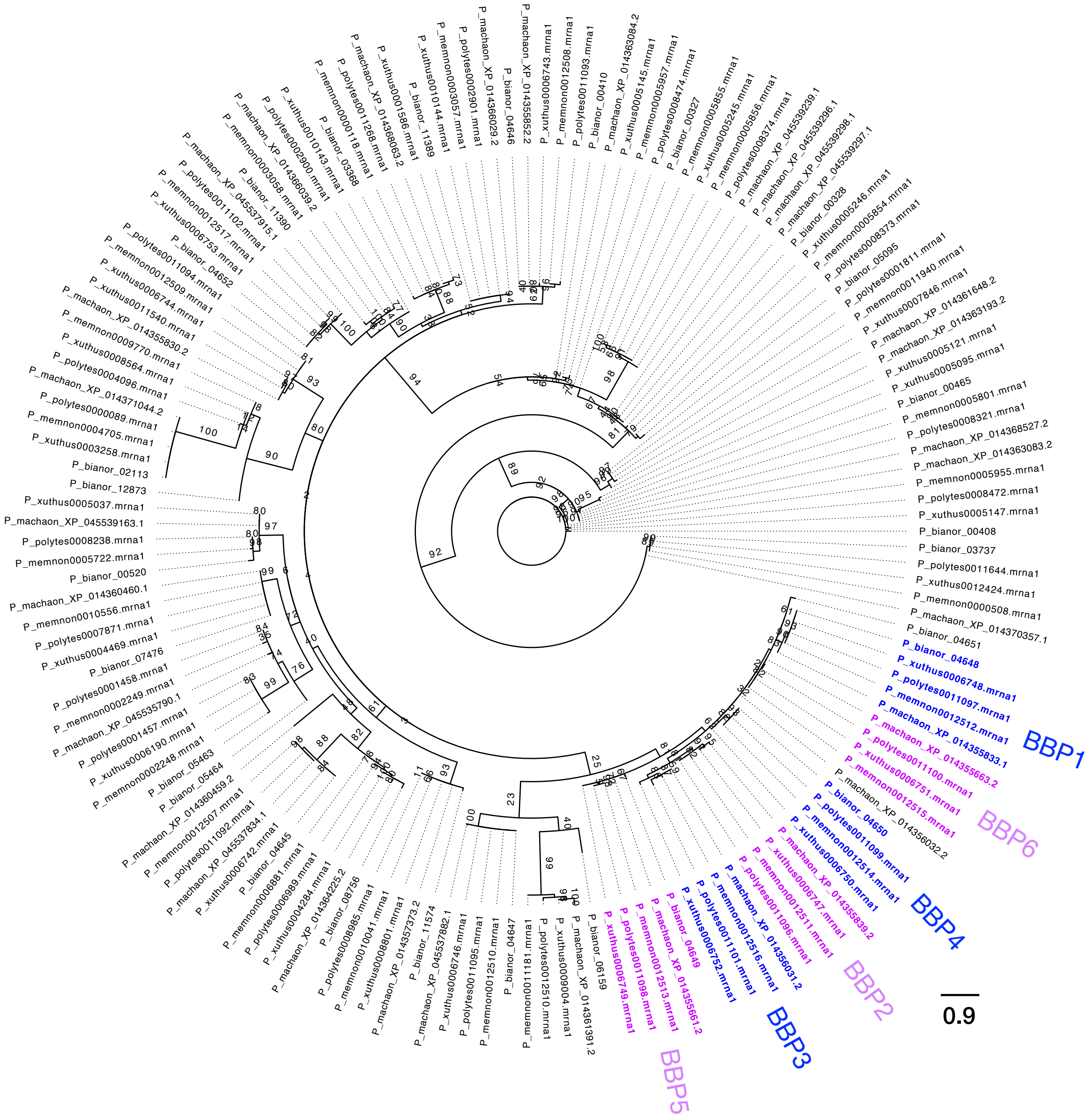

Figure S2. Phylogenetic tree of lipocalin family genes in five *Papilio* butterflies. Unrooted maximum likelihood tree of *BBP*s homologs. Bootstraps values are shown at the tree nodes. The blue and purple clade is *bilin-binding proteins* (*BBP*s). Tree was constructed via the Q.insect+G4 model (automatic selection by IQ-TREE) with 1000 bootstrap numbers. Identifiers and source for all lipocalin family genes are provided in Supplementary Table S5.

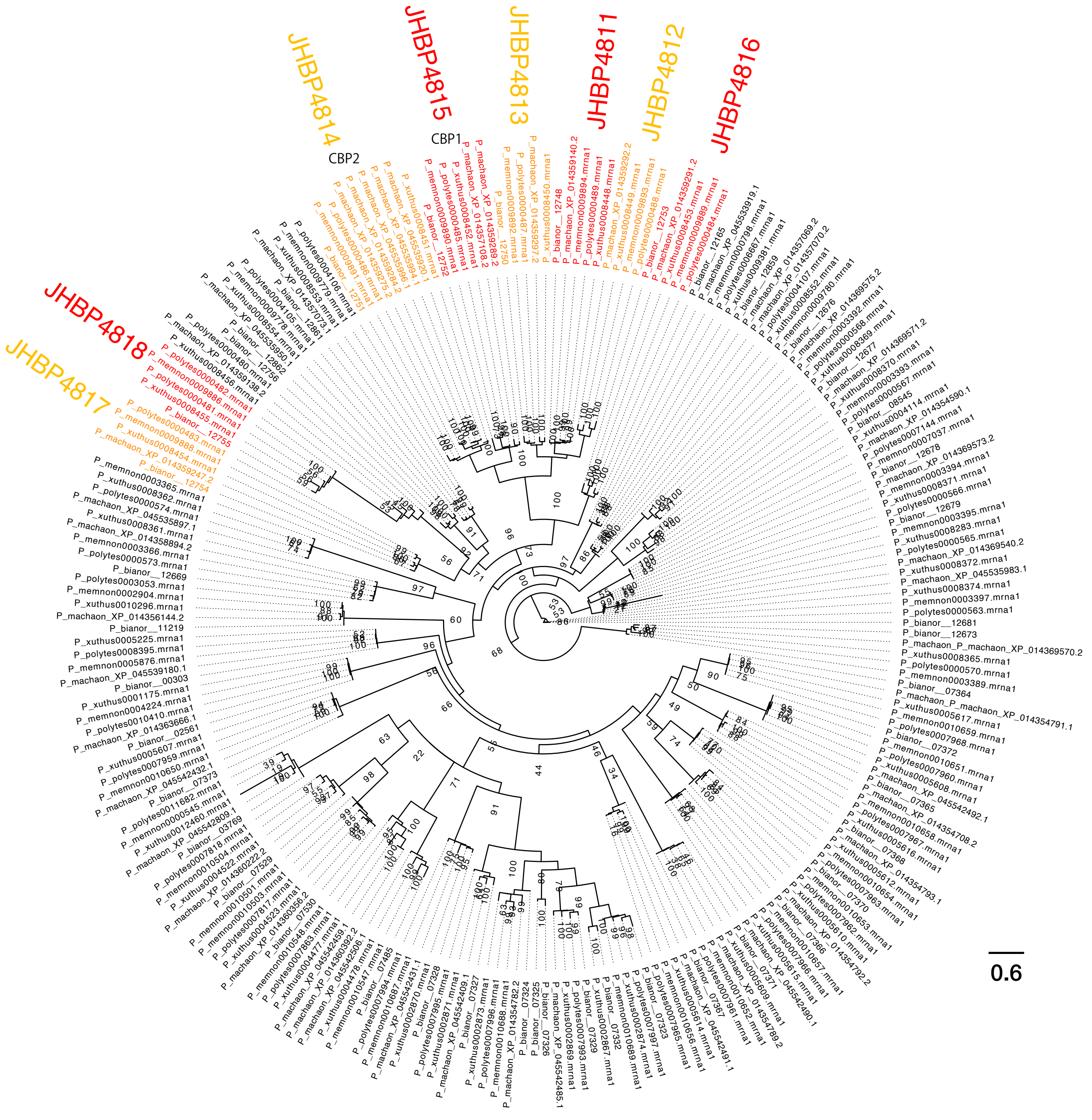

Figure S3. Phylogenetic tree of juvenile hormone binding proteins (JHBPs) in five *Papilio* butterflies. Unrooted maximum likelihood tree of *JHBP*s homologs. Bootstraps values are shown at the tree nodes. The red and orange clades are *JHBP*s in the locus containing *carotenoid binding proteins* (*CBP*s). Tree was constructed via the Q.insect+G4 model (automatic selection by IQ-TREE) with 1000 bootstrap numbers. Identifiers and source for all *JHBP*s are provided in Supplementary Table S6.

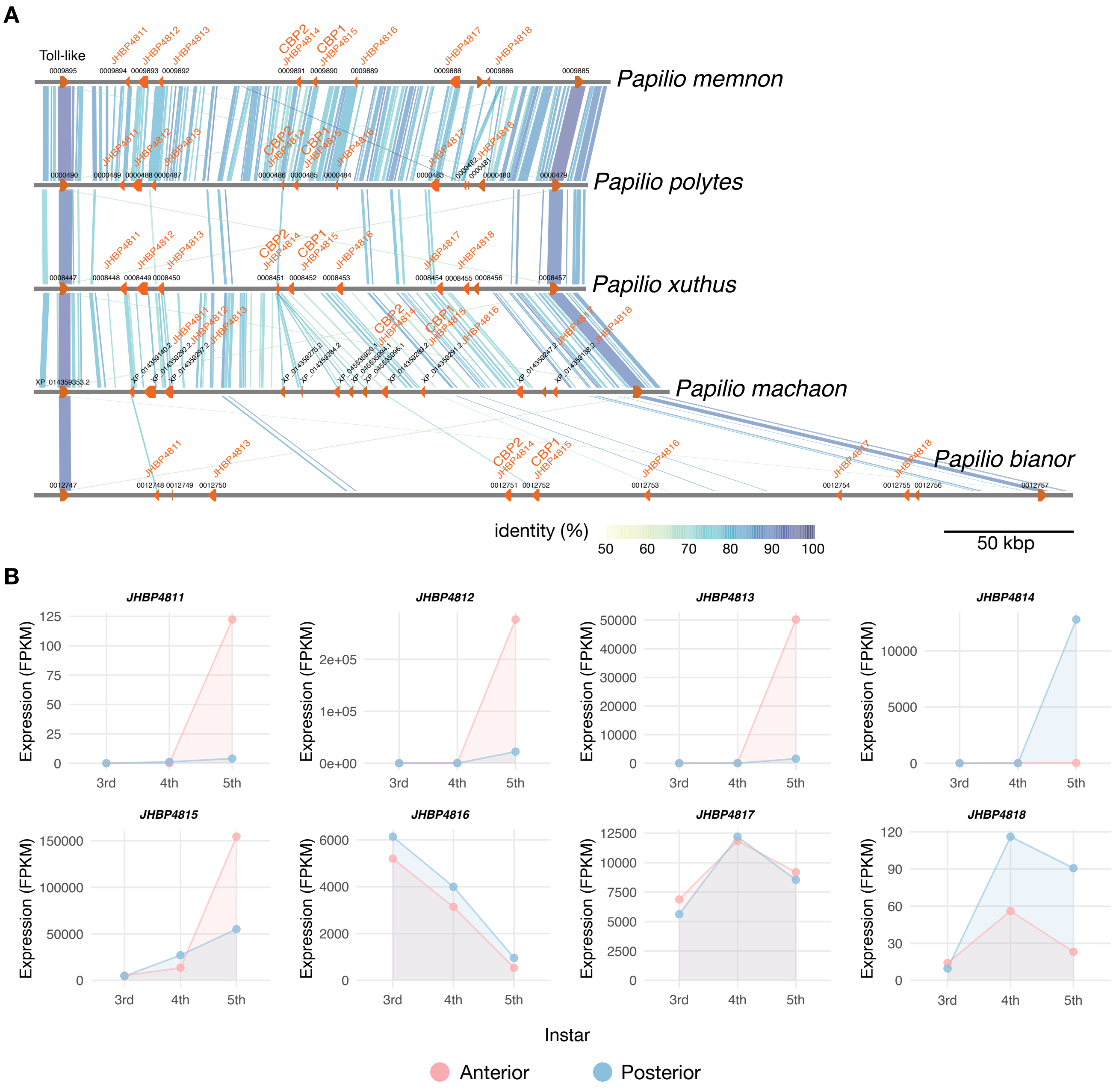

Figure S4. Cluster and expression profiles of *JH-binding proteins* (*JHBPs*). (A) Cluster of *JHBP*s containing *carotenoid binding proteins* (*CBP*s) in 5 *Papilio* species. The length of arrows represents the relative gene length. Transcriptional orientation is shown by the direction of arrows. (B) Expression profiles of *JHBP*s in the locus containing *CBP*s from 3rd- to 5th-instar in epidemics. The expression level of *JHBP*s is shown by the fragments per kilobase of exon per million mapped fragments (FPKM).

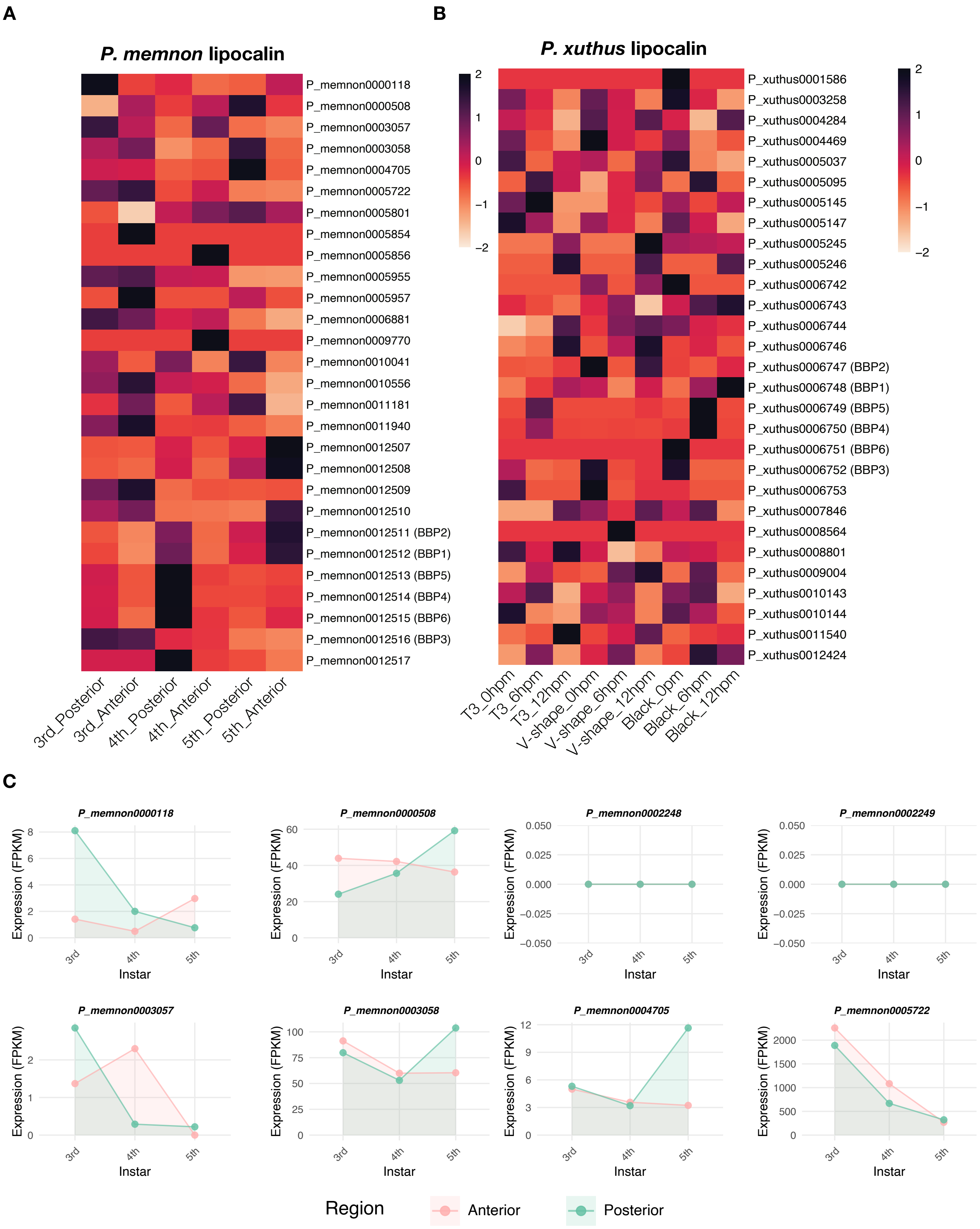

Figure S5. Gene expression profiles of genome-wide *lipocalin* family genes in *Papilio memnon* and *Papilio xuthus* (continued on next page).

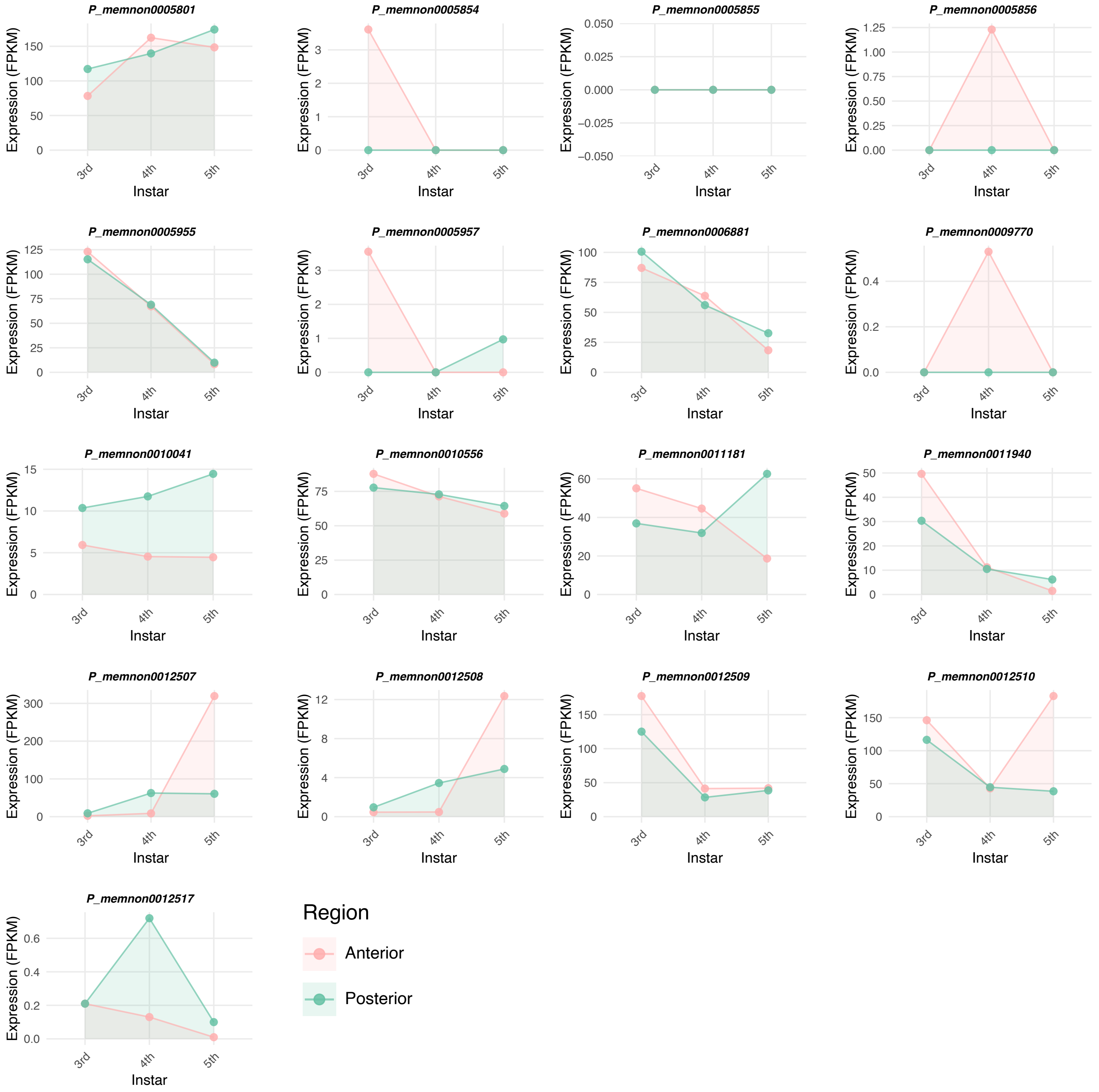

D

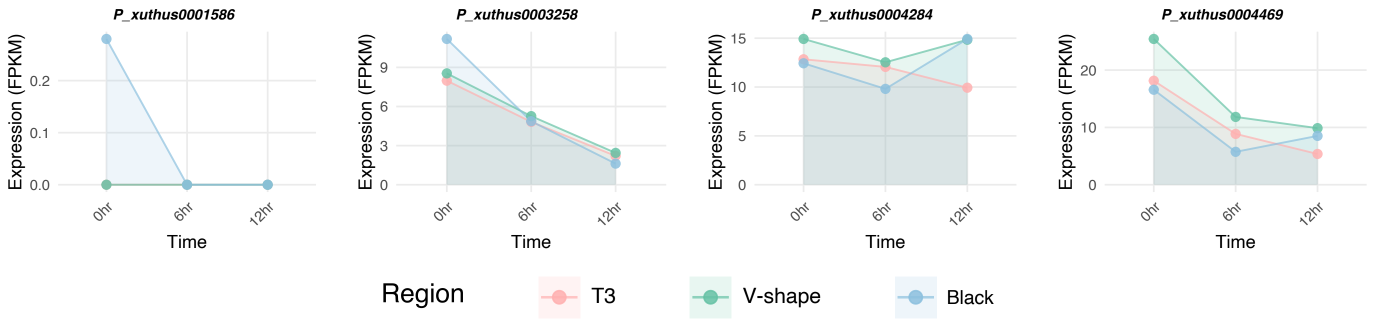

Figure S5. Gene expression profiles of genome-wide *lipocalin* family genes in *Papilio memnon* and *Papilio xuthus* (continued on next page).

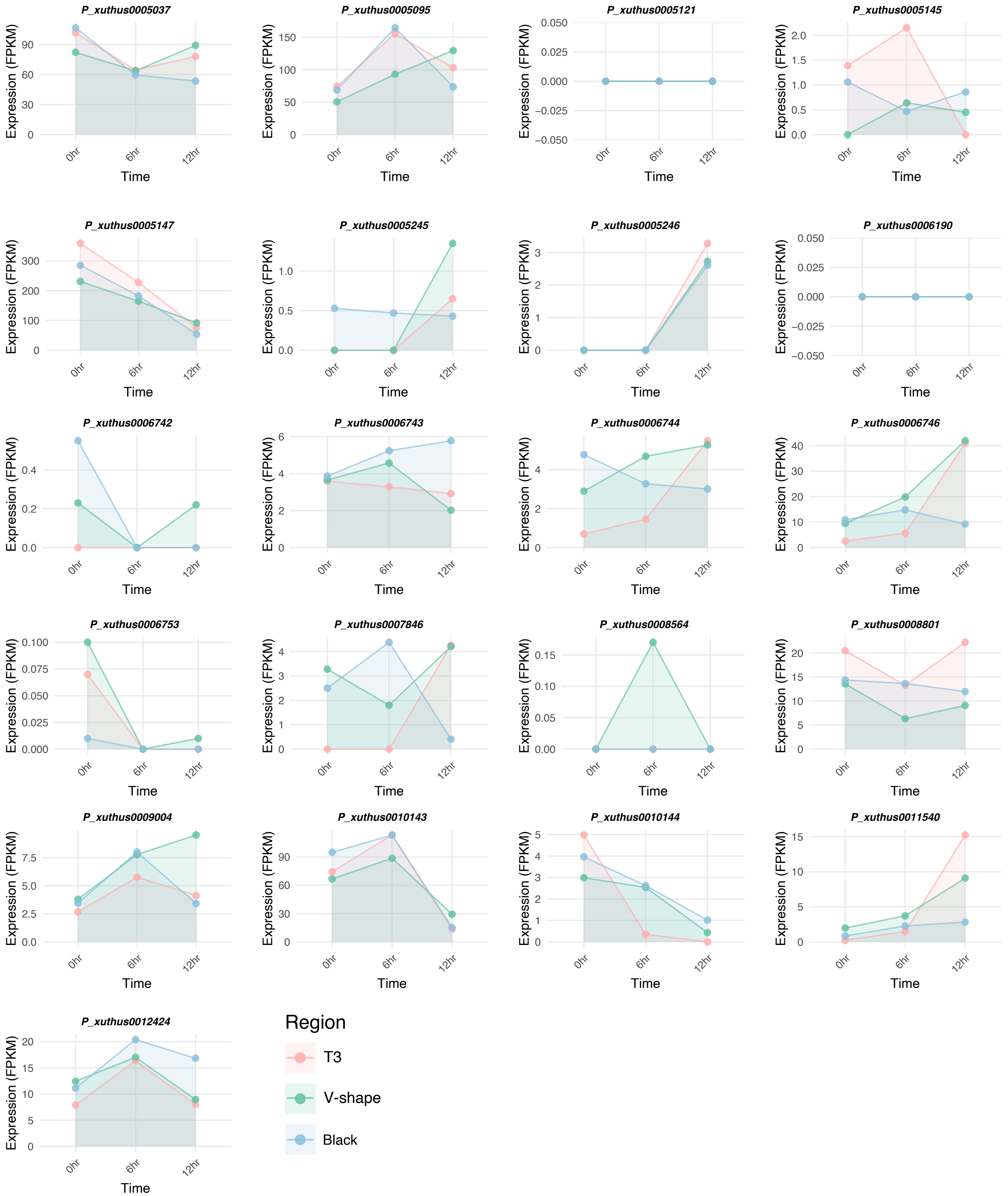

Figure S5. Gene expression profiles of genome-wide *lipocalin* family genes in *Papilio memnon* and *Papilio xuthus*. (A–B) Expression heatmaps of lipocalin family genes in *P. memnon* (A) and *P. xuthus* (B), standardized by z-score normalization across samples. (C–D) Absolute expression levels (FPKM, fragments per kilobase of exon per million mapped fragments) of lipocalin family genes in *P. memnon* (C) and *P. xuthus* (D). For *P. memnon*, “3rd,” “4th,” and “5th” indicate the 3rd, 4th, and 5th larval instars, respectively. For *P. xuthus*, all samples represent 4th instar larvae collected at three time points following the 3rd molt. Hpm: Hours Post the 3rd Molt. T3: the 3rd thoracic segment (eyespot region). V-shape: the putative V-shape region spanning the 4th abdominal segment to the 5th abdominal segment in the 5th instar larvae. Black: the black region spanning the 3rd abdominal and 4th abdominal segments in the 4th instar.

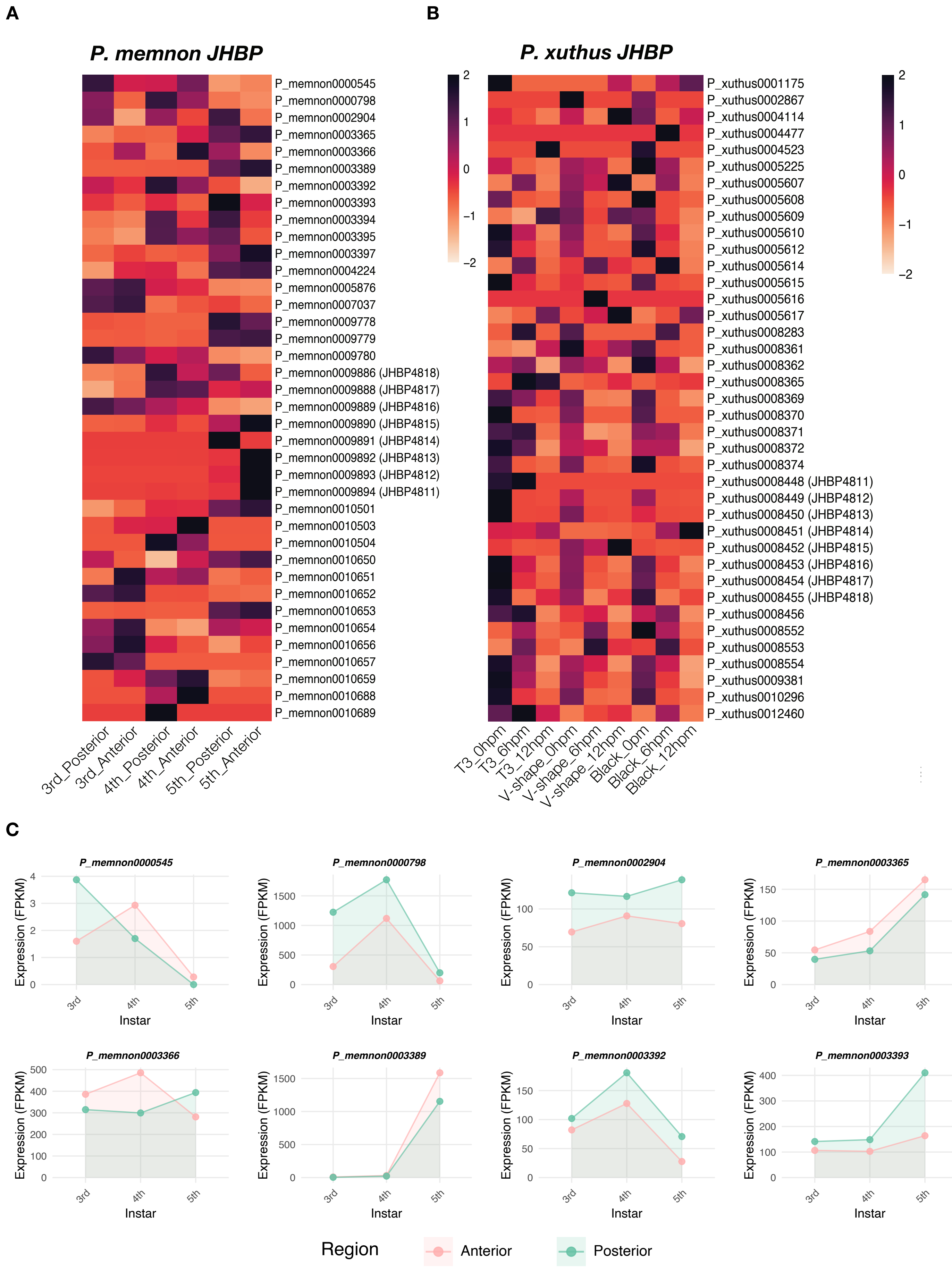

Figure S6. Gene expression profiles of genome-wide JH-binding proteins (*JHBPs*) in *Papilio memnon* and *Papilio xuthus* (continued on next page).

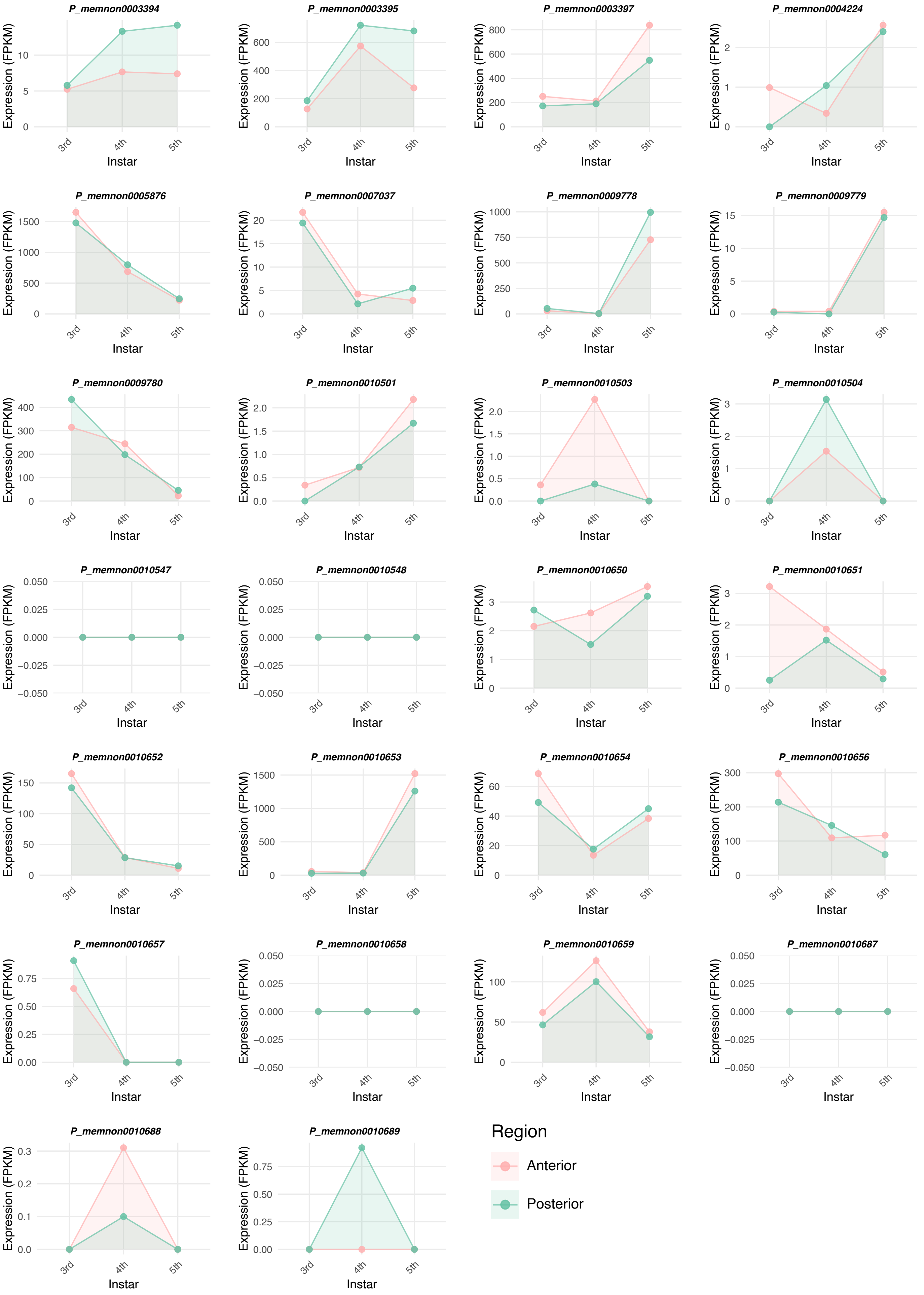

Figure S6. Gene expression profiles of genome-wide JH-binding proteins (*JHBPs*) in *Papilio memnon* and *Papilio xuthus* (continued on next page).

D

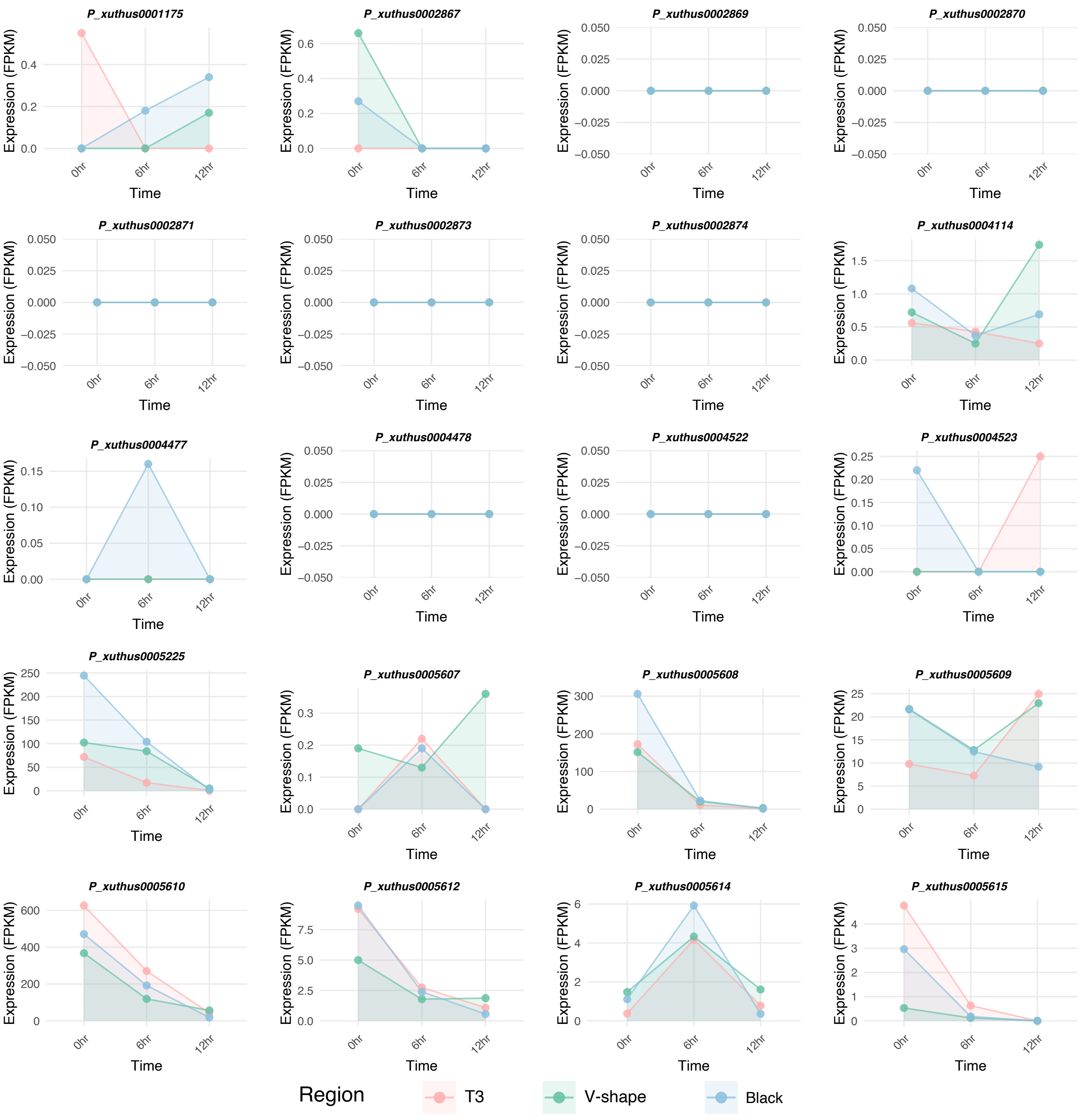

Figure S6. Gene expression profiles of genome-wide JH-binding proteins (JHBPs) in *Papilio memnon* and *Papilio xuthus* (continued on next page). (A–B) Expression heatmaps of JHBP genes in *P. memnon* (A) and *P. xuthus* (B), standardized by z-score normalization across samples. (C–D) Absolute expression 3rd, 4th, and 5th larval instars, respectively. For *P. xuthus*, all samples represent 4th instar larvae expression levels (FPKM, fragments per kilobase of exon per million mapped fragments) of JHBP family genes in *P. memnon* (C) and *P. xuthus* (D). For *P. memnon*, "3rd," "4th," and "5th" indicate the time at three time points following the 3rd molt. Hpm: Hours Post the 3rd Molt. T3: the 3rd thoracic segment (eyespot region). V-shape: the putative V-shape region spanning the 4th abdominal segment to the 5th abdominal segment in the 5th instar larvae. Black: the black region spanning the 3rd abdominal and 4th abdominal segments in the 4th instar.

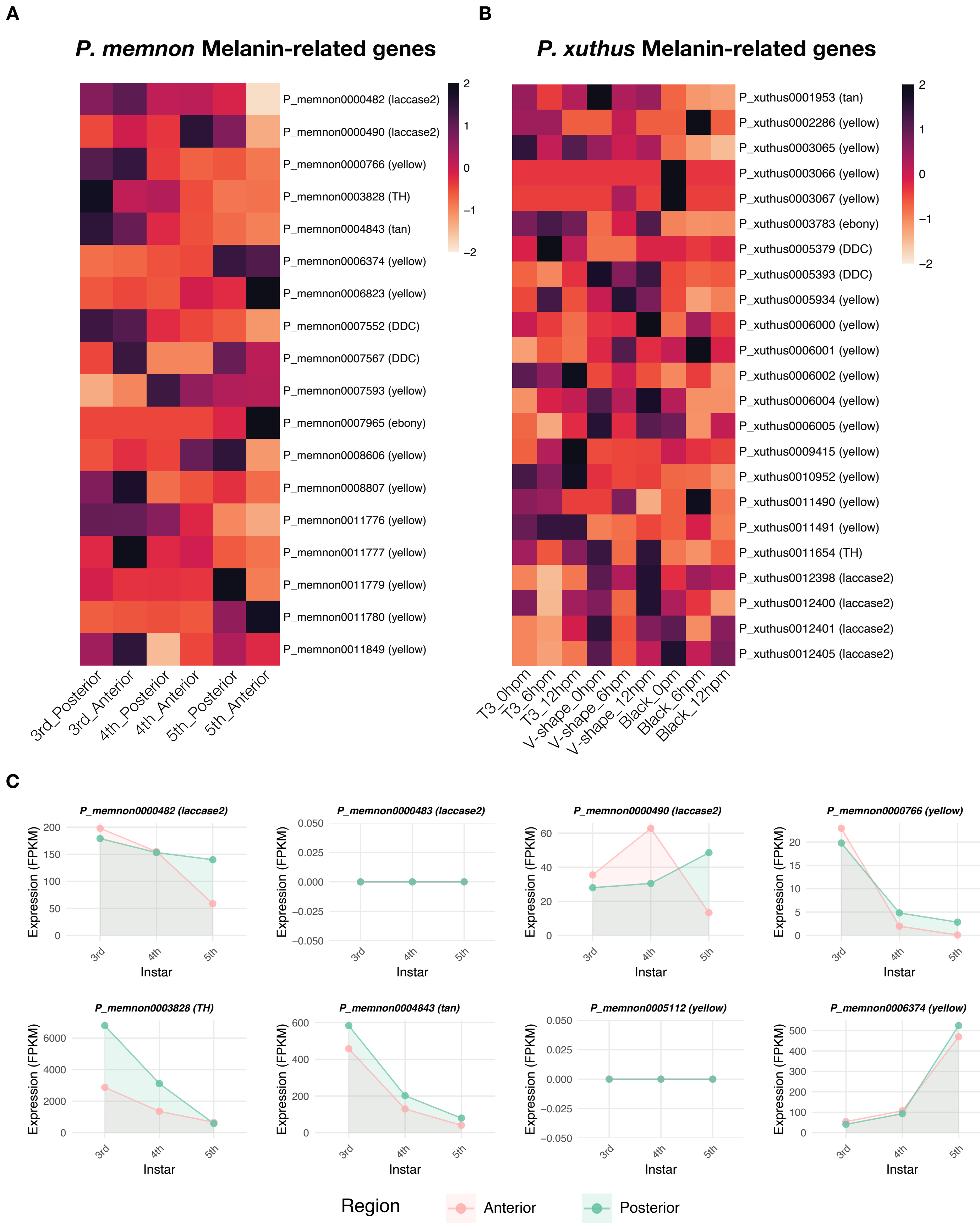

Figure S7. Gene expression profiles of genome-wide melanin-related genes in *Papilio memnon* and *Papilio xuthus* (continued on next page).

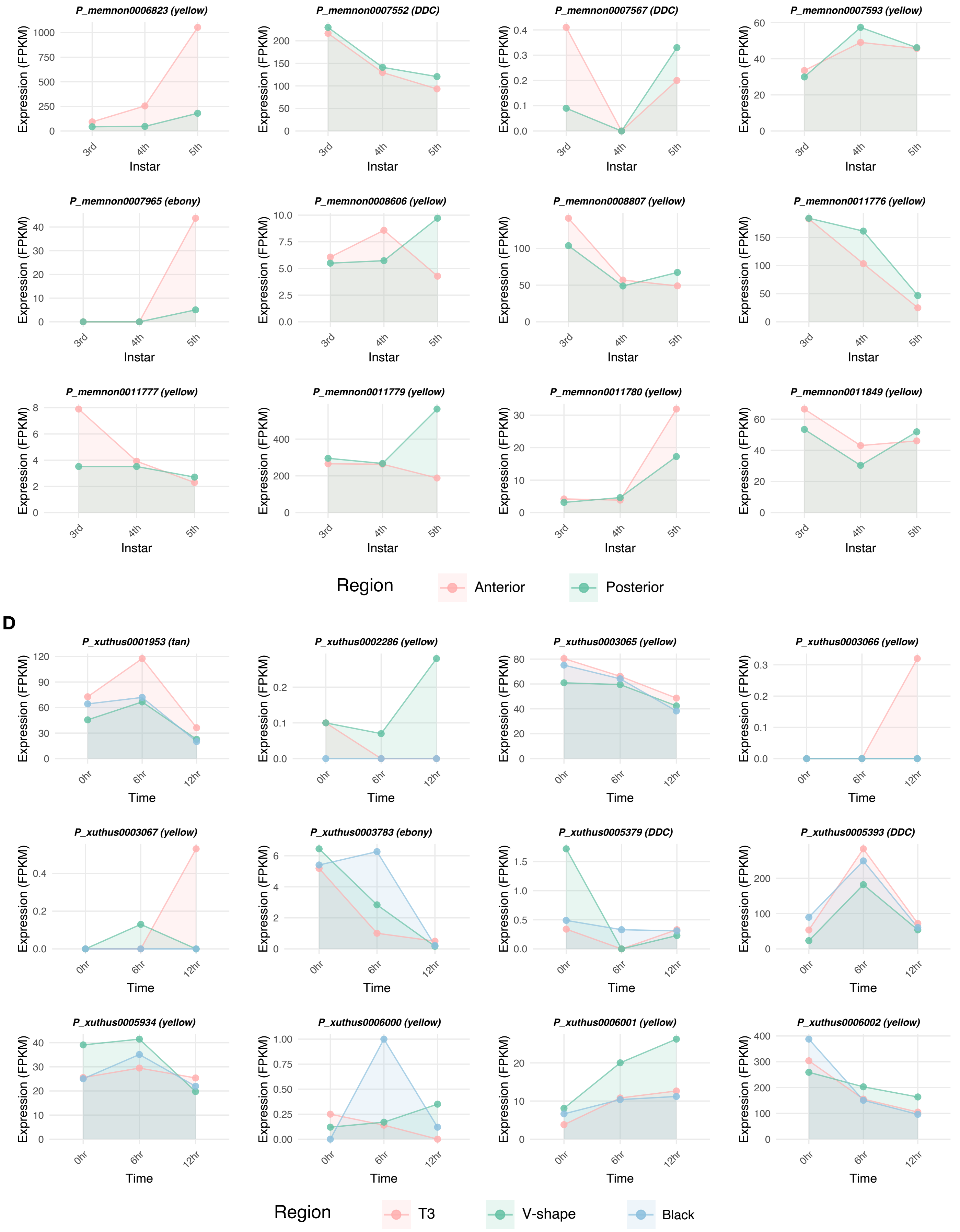

Figure S7. Gene expression profiles of genome-wide melanin-related genes in *Papilio memnon* and *Papilio xuthus* (continued on next page).

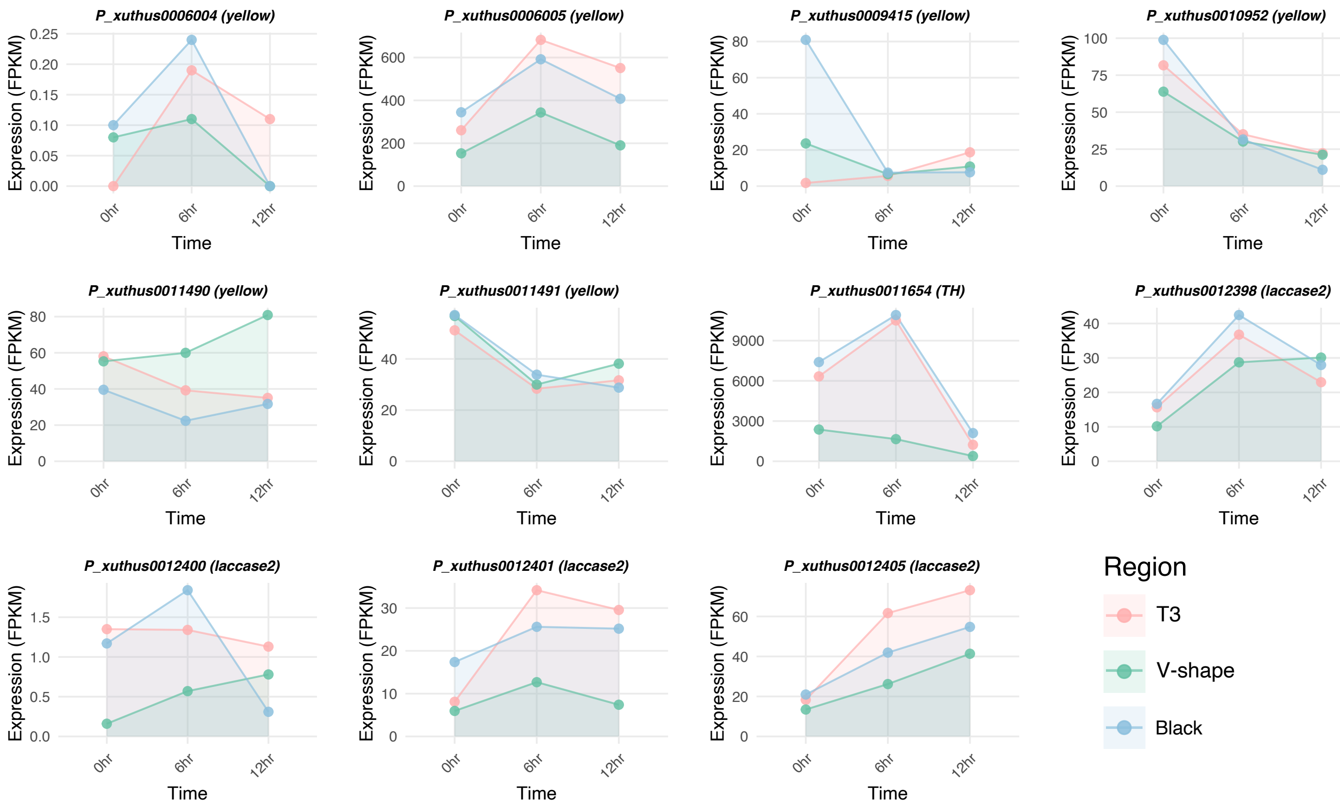

Figure S7. Gene expression profiles of genome-wide melanin-related genes in *Papilio memnon* and *Papilio xuthus.* (A–B) Expression heatmaps of melanin-related genes in *P. memnon* (A) and *P. xuthus* (B), standardized by z-score normalization across samples. (C–D) Absolute expression 3rd, 4th, and 5th larval instars, respectively. For *P. xuthus*, all samples represent 4th instar larvae expression levels (FPKM, fragments per kilobase of exon per million mapped fragments) of melanin-related genes in *P. memnon* (C) and *P. xuthus* (D). For *P. memnon*, "3rd," "4th," and "5th" indicate the time at three time points following the 3rd molt. Hpm: Hours Post the 3rd Molt. T3: the 3rd thoracic segment (eyespot region). V-shape: the putative V-shape region spanning the 4th abdominal segment to the 5th abdominal segment in the 5th instar larvae. Black: the black region spanning the 3rd abdominal and 4th abdominal segments in the 4th instar.

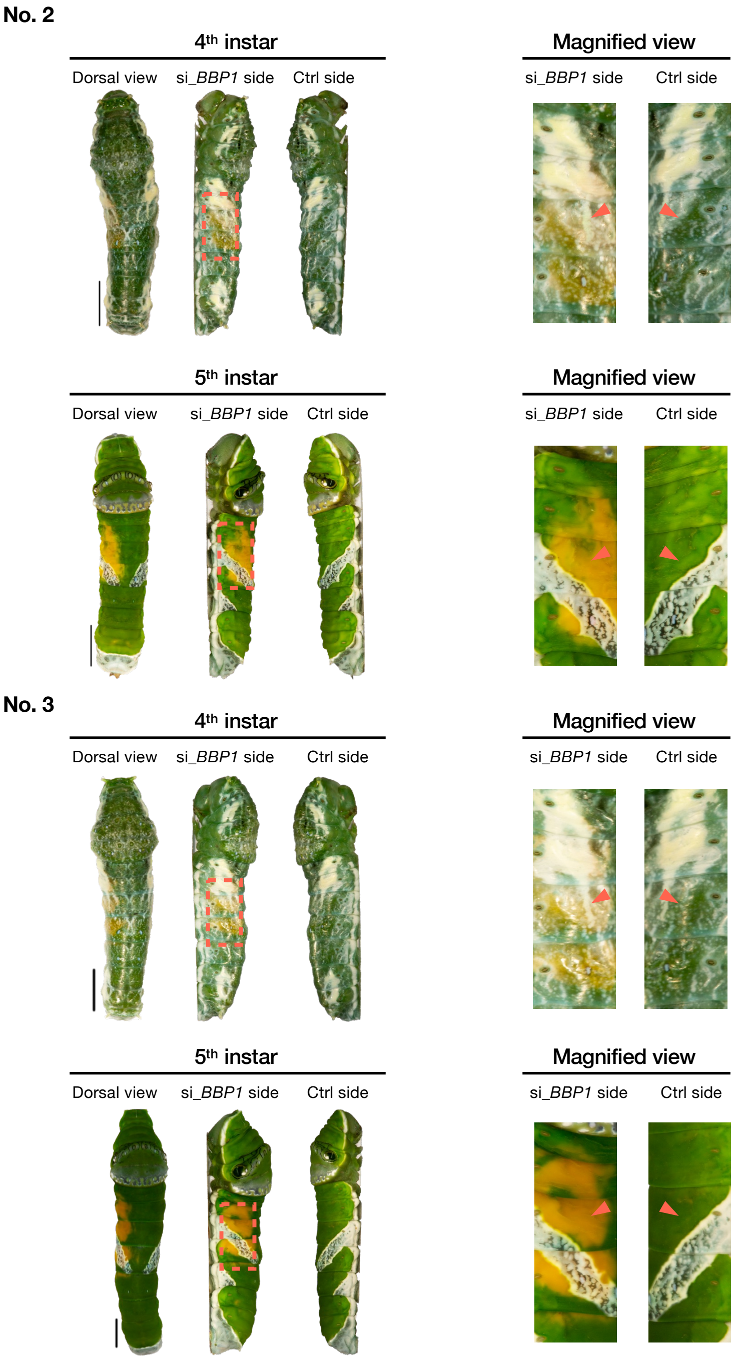

Figure S8. in vivo electroporation-mediated RNAi of *BBP1*. Additional individuals. 4th-instar (left) and 5th-instar (right) larvae after RNAi of *BBP1*. siRNA was injected through the intersegmental membrane between the 7th and 8th abdominal segment and introduced into a specific epidermal region (indicated by red dashed frame) via an electroporation-mediated method during the 3rd instar. scale bar = 5 mm.

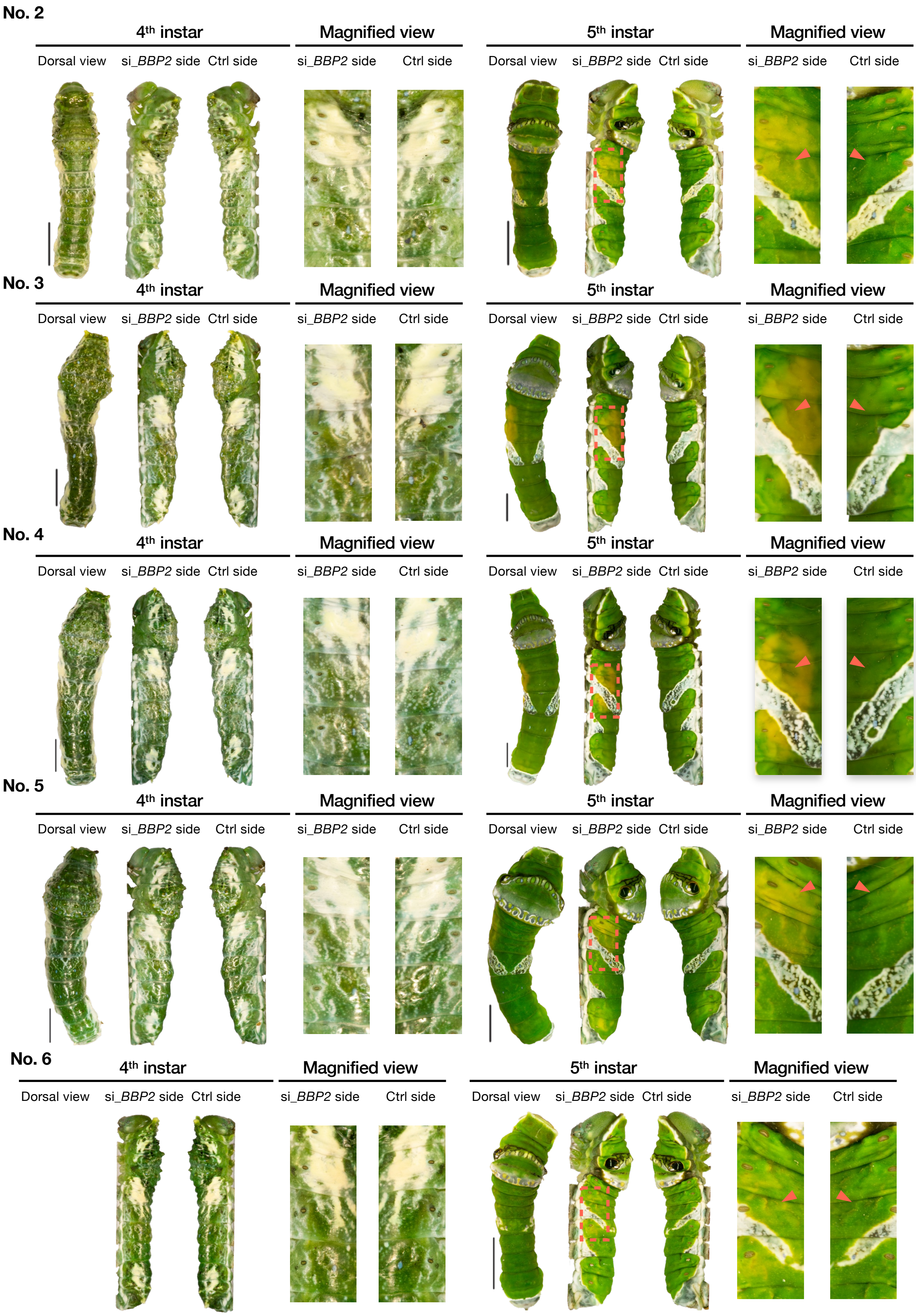

Figure S9. in vivo electroporation-mediated RNAi of *BBP2*. Additional individuals. 4th instar (left) and 5th instar (right) larvae after RNAi of *BBP2*. siRNA was injected through the intersegmental membrane between the 7th and 8th abdominal segment and introduced into a specific epidermal region (indicated by red dashed frame) via an electroporation-mediated method during the 3rd instar. scale bar = 5 mm.

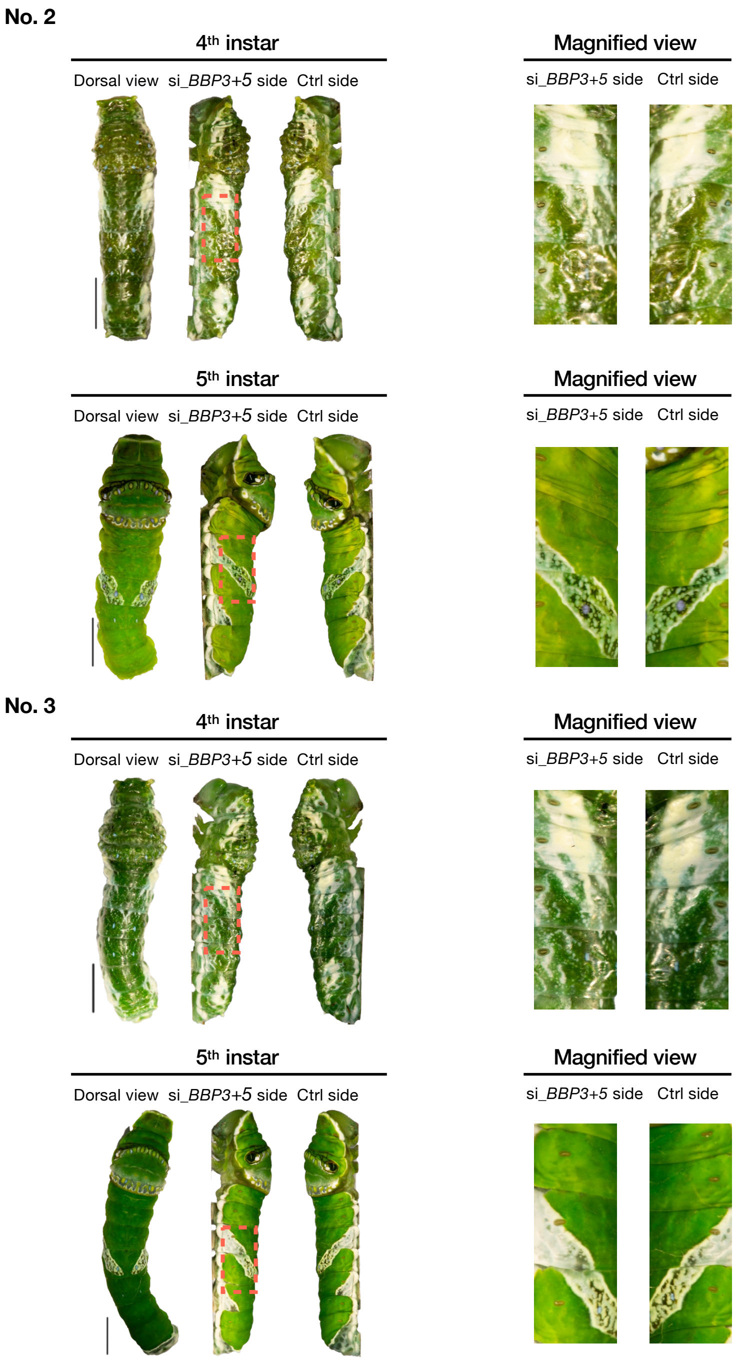

Figure S10. in vivo electroporation-mediated RNAi of *BBP 3 + 5*. Additional individuals. 4th-instar (left) and 5th instar (right) larvae after RNAi of *BBP3 + 5*. siRNA was injected through the intersegmental membrane between the 7th and 8th abdominal segment and introduced into a specific epidermal region (indicated by red dashed frame) via an electroporation-mediated method during the 3rd instar. scale bar = 5 mm.

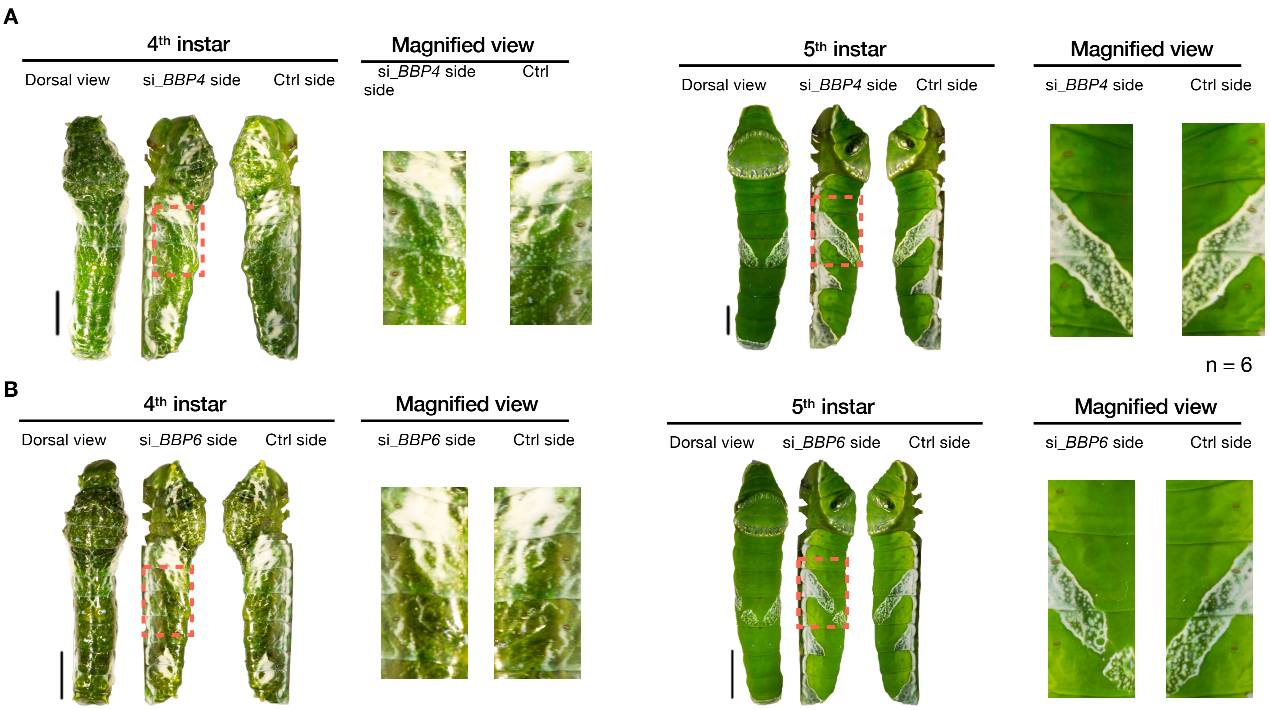

Figure S11. in vivo electroporation-mediated RNAi of *BB4* and *BBP6*. (A) 4th instar (left) and 5th instar (right) larvae after RNAi of *BBP4* (B) 4th instar (left) and 5th instar (right) larvae after RNAi of *BBP6*. siRNA was injected through the intersegmental membrane between the 7th and 8th abdominal segment and introduced into a specific epidermal region (indicated by red dashed frame) via an electroporation-mediated method during the 3rd instar. scale bar = 5 mm. The knockdown of *BBP6* resulted in the elimination of the V-shaped pattern across the 4th and 5th abdominal segments.

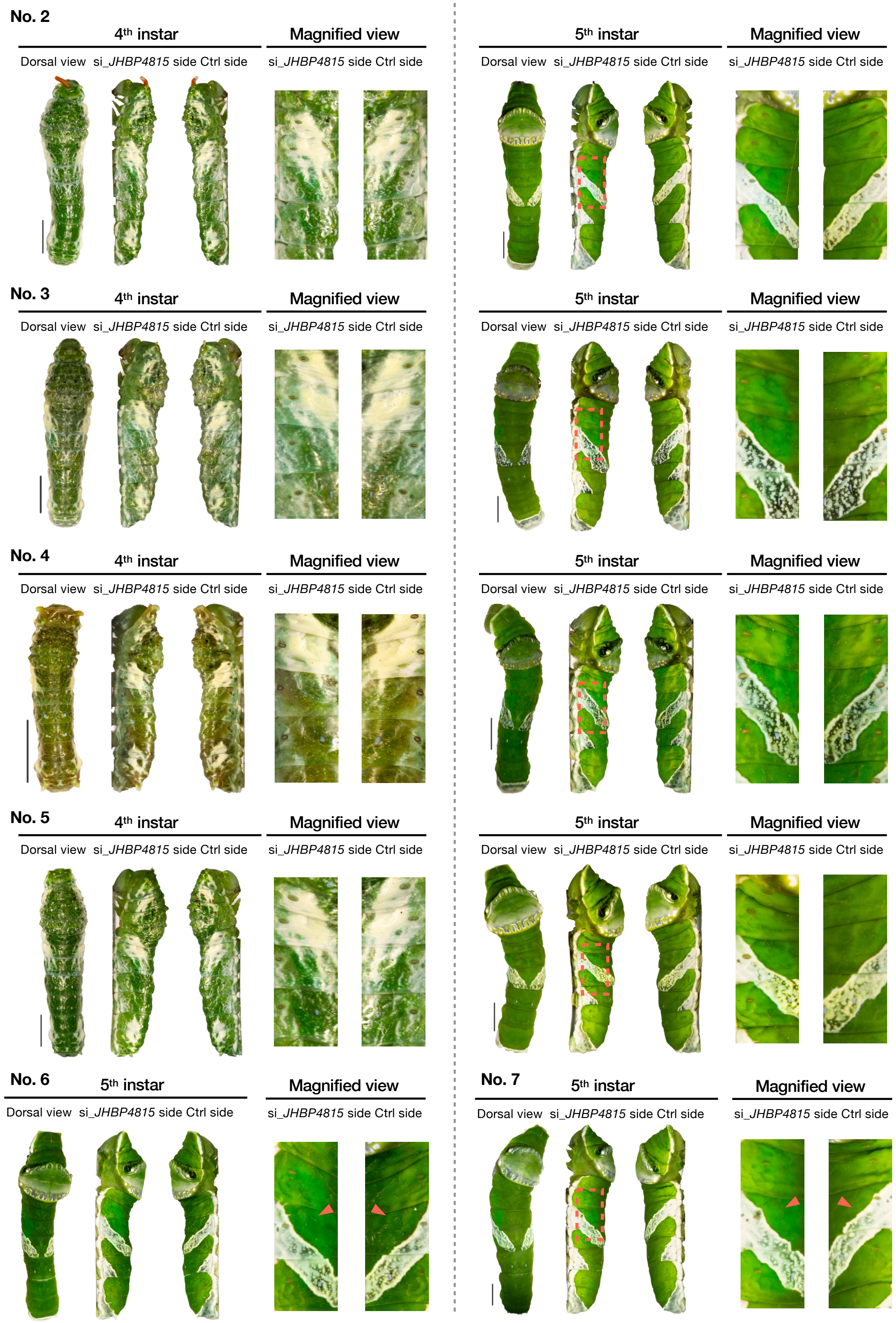

Figure S12. in vivo electroporation-mediated RNAi of *JHBP4815*. Additional individuals. 4th instar (left) and 5th instar (right) larvae after RNAi of *JHBP4815*. siRNA was injected through the intersegmental membrane between the 7th and 8th abdominal segment and introduced into a specific epidermal region (indicated by red dashed frame) via an electroporation-mediated method during the 3rd instar. scale bar = 5 mm.

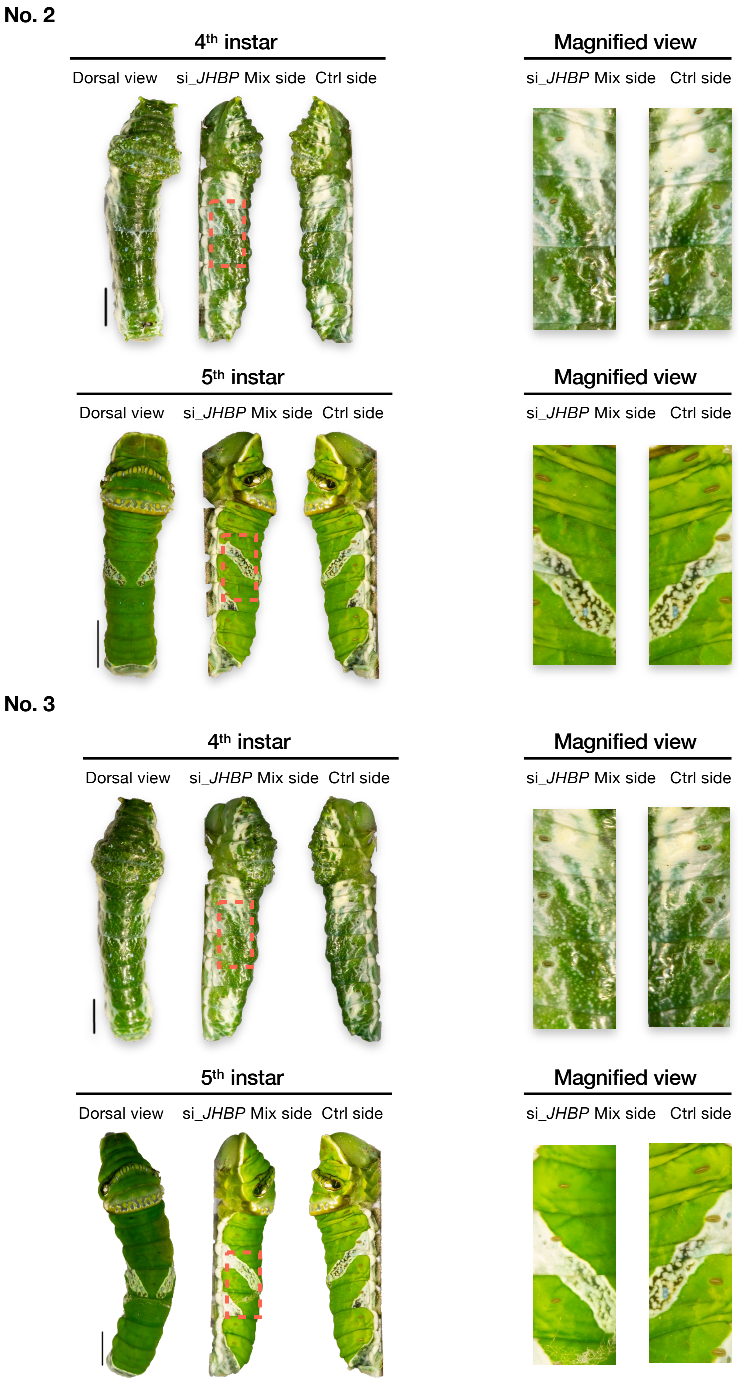

Figure S13. in vivo electroporation-mediated RNAi of *JHBPs*. Additional individuals. 4th instar (left) and 5th instar (right) larvae after multiple-RNAi of *JHBP4812, JHBP4814, JHBP4816* and *JHBP2505* (an additional JHBP family gene located adjacent to the JHBP cluster). siRNA was injected through the intersegmental membrane between the 7th and 8th abdominal segment and introduced into a specific epidermal region (indicated by red dashed frame) via an electroporation-mediated method during the 3rd instar. scale bar = 5 mm. Among all the RNAi experiments targeting *JHBPs*, only knockdown of *JHBP4815* caused a slight reduction of yellow color during the larval stage.

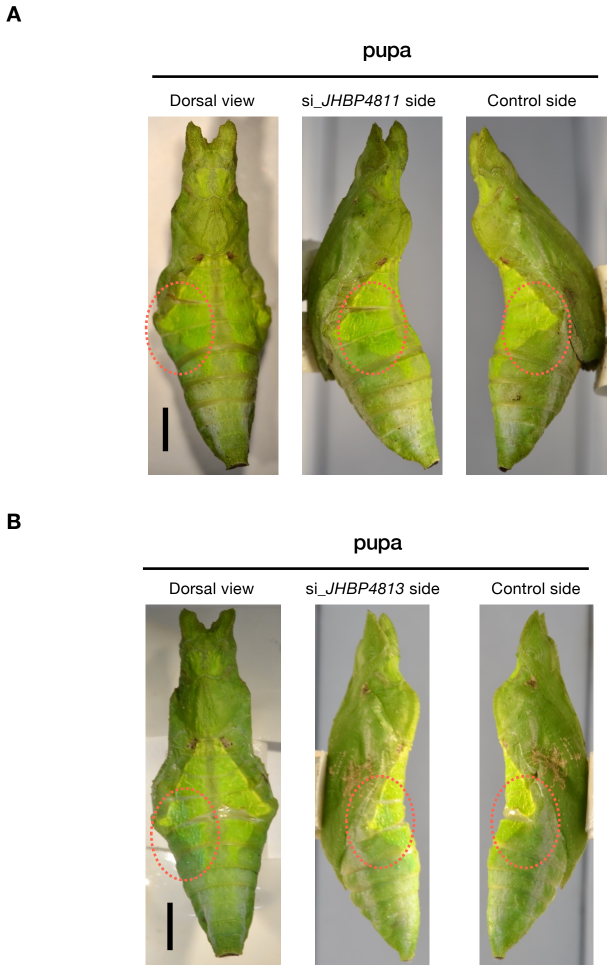

Figure S14. Electroporation-mediated RNAi of *JHBP4811* and *JHBP4813*. (A) pupa after the RNAi of *JHBP4811, n* = 4. (B) pupa after the RNAi of *JHBP4813, n* = 2. siRNA was injected through the intersegmental membrane between the 7th and 8th abdominal segment and introduced into a specific epidermal region (indicated by red dashed frame) via an electroporation-mediated method during the 3rd instar. scale bar = 5 mm.

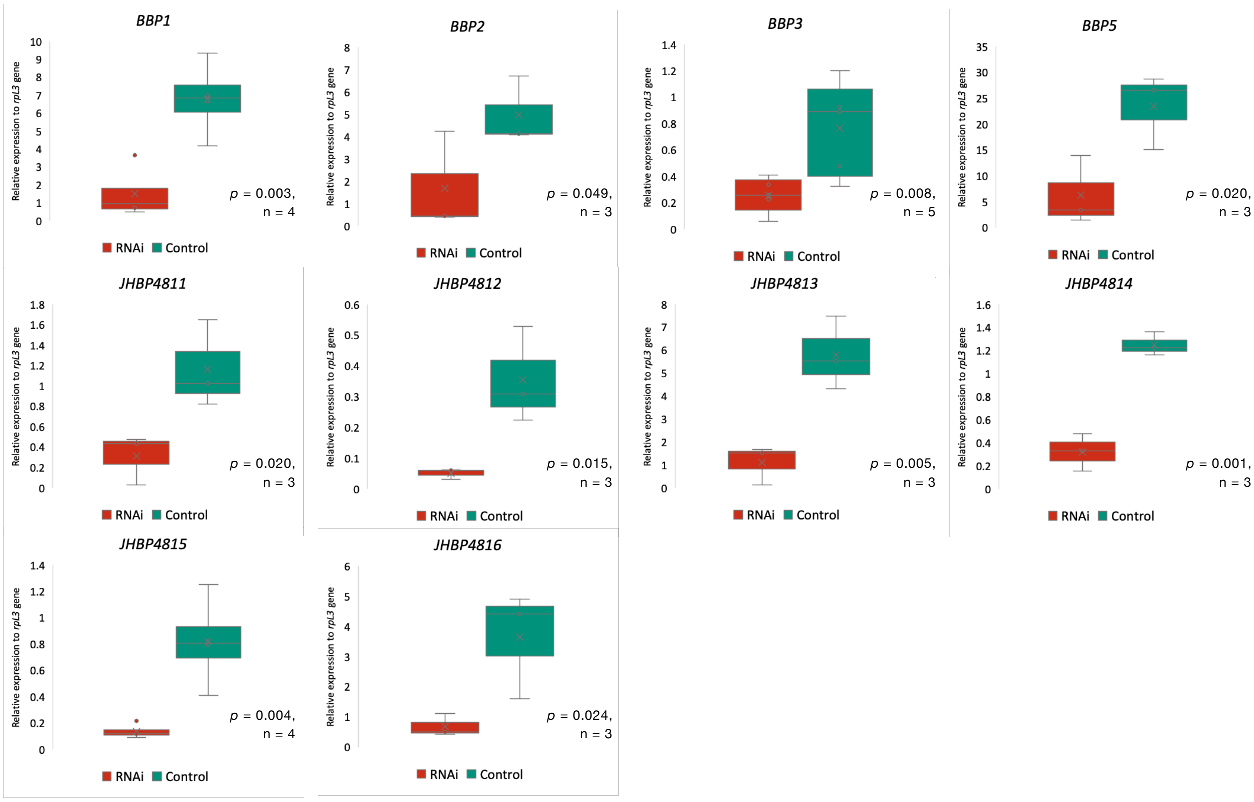

Figure S15. Knockdown efficiency of RNAi experiments by qPCR. *ribosomal protein L3* (*rpL3*) was used as a internal reference gene. Relative expressions of the RNAi and control sides were plotted in red and green colors. Statistical significance was examined using Student's *t*-test. *p*-values and sample sizes number of individuals are indicated in the plot. Primers used for qPCR in this study are listed in Table S6.

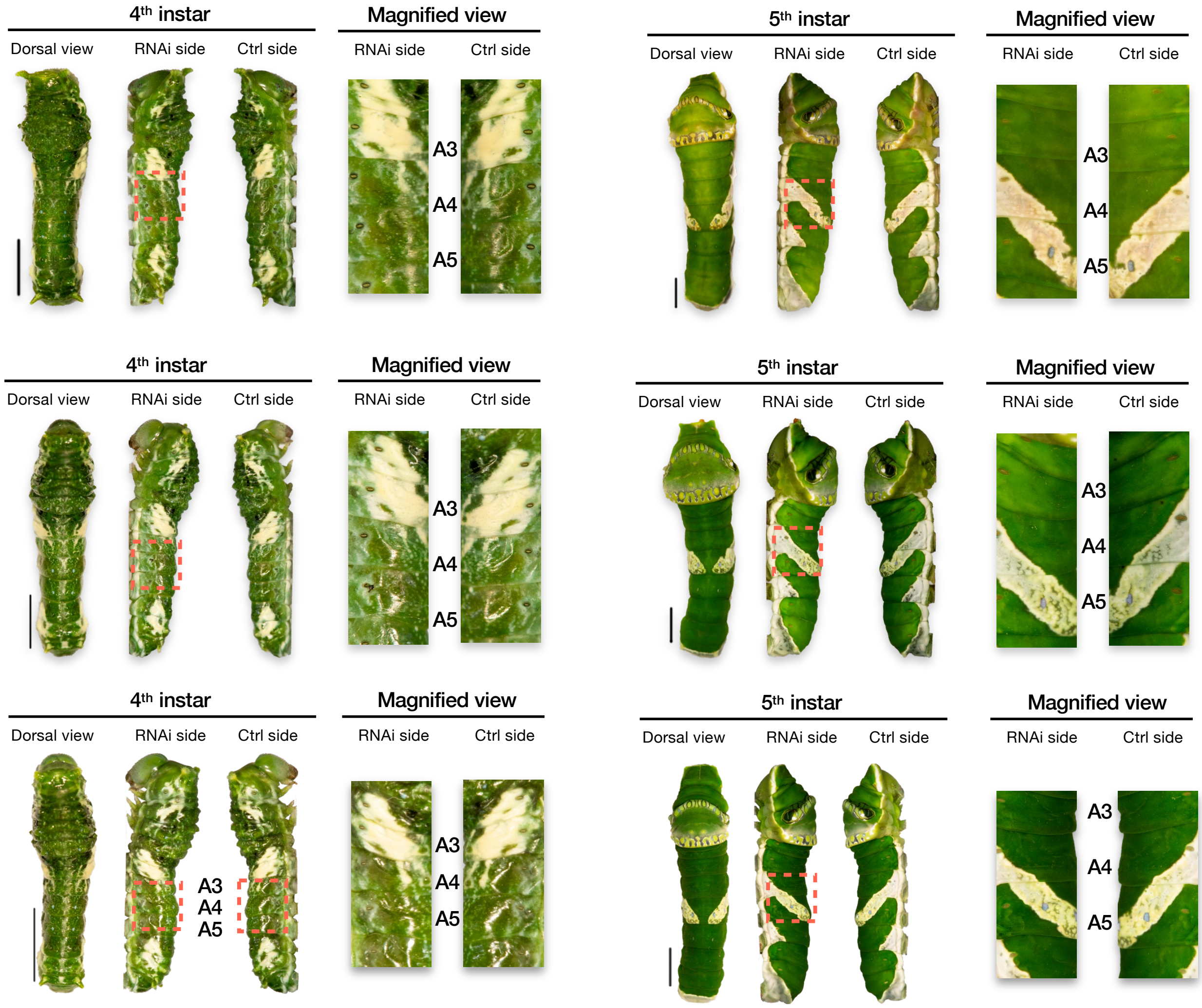

Figure S16. Negative Control. Left panel, the phenotype of the 4th instar larva after the electroporation. Right panel, the phenotype of the 5th instar larva after the electroporation. 1 µl 250 mM universal siRNA was injected at the late 3rd instar stage through the intersegmental membrane between the 7th and 8th abdominal segment and introduced into a specific epidermal region (indicated by red dashed frame) via an electroporation-mediated method. scale bar = 5 mm. A3, A4 and A5: the 3rd, 4th and 5th abdominal segments, respectively. n = 3.

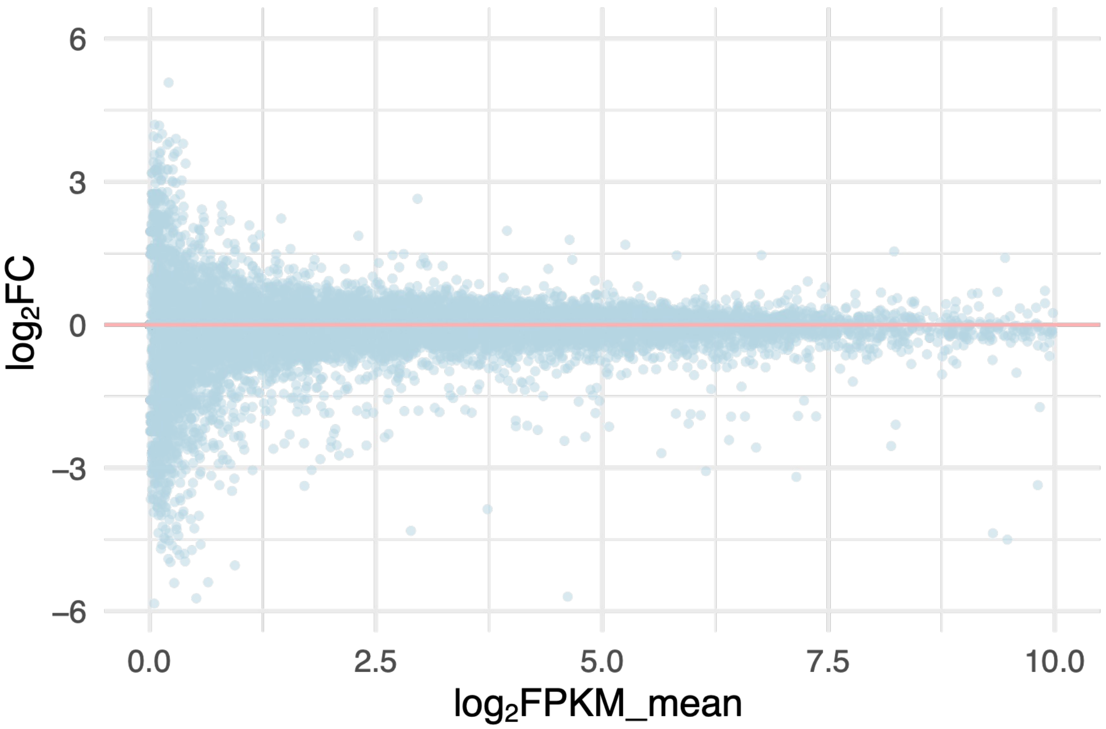

Figure S17. MA plot of gene expression across larval samples. Each point represents a gene, plotted by mean expression level (log_2FPKM_mean) on the x-axis and log_2 fold change (log2FC) on the y-axis. The horizontal pink line indicates $log_2FC = 0$.

Table S1. Statistics of protein-coding genes. *Papilio memnon*, *Papilio polytes* and *Papilio xuthus* are annotated in this study.

Species	Genes	Total gene length	Mean gene length	Exons	Mean exons/mrna	Single exon gene	Total exon length	Mean exon length	Mean cds length	Intron	Total intron length	Mean intron length
Papilio memenon (annotated in this study)	12,943	108,970,781	8,419.20	93,855	7.25	1,669	20,582,426	219.3	1,590.20	80,912	88,388,355	1092.4
Papilio polytes (annotated in this study)	12,512	110,891,542	8,862.82	92,642	7.4	1,580	20,767,782	224.17	1,659.81	80,130	90,123,760	1,124.72
Papilio xuthus (annotated in this study)	12,917	102,506,041	7,935.75	95,425	7.39	1,520	20,848,159	218.48	1,613.97	82,508	81,657,882	989.7
Papilio machaon (NCBI Refseq annotation (GCF_912999745.1)	13,885	297,336,464	21,414	282,435	10.3	1,289	89,236,500	315	2,071	240,882	627,009,358	2,602
Papilio bianor (Lu et al., 2019)	15,375	136,940,518	8,906	98,231	6.4	1,013	21,270,770	216	1,383	82,856	115,669,748	1,396

Table S2. BUSCO (v5.4.3) statistics with lepidoptera_odb10 database of the gene annotation.

Species	Complete (%)	Single complete (%)	Duplicated complete (%)	Fragmented (%)	Missing (%)
Papilio memnon	97.6	97.2	0.4	0.2	2.2
Papilio polytes	96.9	96.2	0.7	0.3	2.8
Papilio xuthus	97.7	97.7	0.2	0.3	1.8
Papilio machaon	99.6	78.4	21.2	0.1	0.3
Papilio bianor	90.9	90.3	0.6	2	7.1

Table S3. Statistics of RNA-seq raw data.

Sample name	Read counts	Read base pair (bp)	GC content
3rd-Anterior	53,723,644	5,426,088,044	33.57%
3rd-Posterior	50,693,406	5,120,034,006	39.56%
4th-Anterior	54,697,212	5,524,418,412	35.8%
4th-Posterior	43,657,636	4,409,421,236	37.7%
5th-Anterior	41,849,038	41,849,038	42.07%
5th-Posterior	45,115,144	4,556,629,544	41.32%

Table S4. Genes highly expressed in the green region of *Papilio memnon* larvae.

Shown in excel file.

Table S5. Lipocalin family genes in five *Papilio* butterflies.

Shown in excel file.

Table S6. Juvenile hormone binding proteins (JHBPs) in five *Papilio* butterflies.

Shown in excel file.

Table S7. siRNA list.

Target gene	siRNA name		Sequence
Bilin-binding protein 1	*PmeBBP1*	Target (DNA)	AGGTTATGCTGACATACACATTT
		Antisense strand (RNA)	AUGUGUAUGUCAGCAUAACCU
		Sense strand (RNA)	GUUAUGCUGACAUACACAUUU
Bilin-binding protein 2	*PmeBBP2*	Target (DNA)	GAGGAGATAACCGAATTCTTAAA
		Antisense strand (RNA)	UAAGAAUUCGGUUAUCUCCUC
		Sense strand (RNA)	GGAGAUAACCGAAUUCUUAAA
Bilin-binding protein 3	*Pme_BBP3*	Target (DNA)	AAGTAGTGAACGGCGTCAAATCT
		Antisense strand (RNA)	AUUUGACGCCGUUCACUACUU
		Sense strand (RNA)	GUAGUGAACGGCGUCAAAUCU
Bilin-binding protein 4	*Pme_BBP4*	Target (DNA)	AACTTTGATTTCAATGCTTATCA
		Antisense strand (RNA)	AUAAGCAUUGAAAUCAAAGUU
		Sense strand (RNA)	CUUUGAUUUCAAUGCUUAUCA
Bilin-binding protein 5	*Pme_BBP5*	Target (DNA)	AACAGAACTTCGATTTTGCATCT
		Antisense strand (RNA)	AUGCAAAAUCGAAGUUCUGUU
		Sense strand (RNA)	CAGAACUUCGAUUUUGCAUCU
Bilin-binding protein 6	*Pme_BBP6*	Target (DNA)	CTCTAATCGAGTACTACGTATAT
		Antisense strand (RNA)	AUACGUAGUACUCGAUUAGAG
		Sense strand (RNA)	CUAAUCGAGUACUACGUAUAU
JH-binding protein 4811	*Pme_JHBP4811*	Target (DNA)	TTGAATCTACTGGCAAATACAAG
		Antisense strand (RNA)	UGUAUUUGCCAGUAGAUUCAA
		Sense strand (RNA)	GAAUCUACUGGCAAAUACAAG

Continued Table S7. siRNA list.

Target gene	siRNA name		Sequence
JH-binding protein 4812	*Pme_JHBP4812*	Target (DNA)	AAGCGTTACTGGAAACTGAATCT
		Antisense strand (RNA)	AUUCAGUUUCCAGUAACGCUU
		Sense strand (RNA)	GCGUUACUGGAAACUGAAUCU
JH-binding protein 4813	*Pme_JHBP4813*	Target (DNA)	CTCTACTATGTCCGTAACTTTAG
		Antisense strand (RNA)	AAAGUUACGGACAUAGUAGAG
		Sense strand (RNA)	CUACUAUGUCCGUAACUUUAG
JH-binding protein 4814	*Pme_JHBP4814*	Target (DNA)	GTGATGAATCTGCTATGAGATTC
		Antisense strand (RNA)	AUCUCAUAGCAGAUUCAUCAC
		Sense strand (RNA)	GAUGAAUCUGCUAUGAGAUUC
JH-binding protein 4815	*Pme_JHBP4815*	Target (DNA)	CTCCTAAGTTTCGAATACTCAAG
		Antisense strand (RNA)	UGAGUAUUCGAAACUUAGGAG
		Sense strand (RNA)	CCUAAGUUUCGAAUACUCAAG
JH-binding protein 4816	*Pme_JHBP4816*	Target (DNA)	TACCTTCAACGATCTAAACATAT
		Antisense strand (RNA)	AUGUUUAGAUCGUUGAAGGUA
		Sense strand (RNA)	CCUUCAACGAUCUAAACAUAU

Table S8. Electroporation conditions for the *in vivo* RNAi.

	Poring Pulse	Transfer Pulse
Voltage	20 V	15 V
Pulse Length	5 msec	90 msec
Pulse Interval	20 msec	50 msec
Number of Pulse	2	20

Table S9. Primer s for qPCR. (Continued on next page)

Target gene	Primer name	Sequence
ribosomal protein L3	*P. me rpL3* F	CTTGAGAAGCCTATCCCAGTGG
	P. me rpL3 R	GAGCTTTTTAGTGTGCCAACGG
Bilin-binding protein 1	*P. me_BBP1*_F	TACGGTGGGACATGGTACGA
	*P. me_BBP1*_R	TGAATTCCTCGCTTTGCCCT
Bilin-binding protein 2	*P. me_BBP2*_F	AATCGCCAAATTCCCAAACCC
	*P. me_BBP2*_R	CTTGCGCCATCAACTACGTG
Bilin-binding protein 3	*P. me_BBP3*_F	GGTAAATGCTCCACTGCGGA
	*P. me_BBP3*_R	TCCAGGTCCCACAAGAGTCA
Bilin-binding protein 4	*P. me_BBP4*_F	TGAAGCCGATGGAGAACTTTGA
	*P. me_BBP4*_R	GTGTTTCTAACTTTGCCGCTGT
Bilin-binding protein 5	*P. me_BBP5*_F	CCTGTCCGTCTGTCAGACCT
	*P. me_BBP5*_R	CAATCGTGCACTTTCCCCTG
Bilin-binding protein 6	*P. me_BBP6*_F	TTGTTCGTGTTTGCTCTGATCG
	*P. me_BBP6*_R	ATGTTCCTTGGTAAGCGGAGAA
JH-binding protein 4811	*P. me_JHBP4811*_F	CCGAATATGAAGTTAACCGGCG
	*P. me_JHBP4811*_R	CCAGTAGATTCAAACGGACAGTC
JH-binding protein 4812	*P. me_JHBP4812*_F	TTCTTTAGCCCGTCGACTGG
	*P. me_JHBP4812*_R	TGGTACACGTTGGTAGAATGCA
JH-binding protein 4813	*P. me_JHBP4813*_F	GCCCTCTCTAGAATCGGTTATGG
	*P. me_JHBP4813*_R	AGTTACGGACATAGTAGAGCGTG
JH-binding protein 4814	*P. me_JHBP4814*_F	TCCCACCGACAGATCCGAT
	*P. me_JHBP4814*_R	ATTCAACGACCCTGCATCTCC

Continued Table S9. Primer s for qPCR.

Target gene	Primer name	Sequence
JH-binding protein 4815	*P. me_JHBP4815*_F	TGTCGTCAACAACTCCAGCA
	*P. me_JHBP4815*_R	ATTCCCTCGGCGATGTATGG
JH-binding protein 4816	*P. me_JHBP4816*_F	ATAACATCCAGTGCTACGGC
	*P. me_JHBP4816*_R	TTTCATGTCACCCTGGTCGG
ribosomal protein L3	*P. me rpL3* F	CTTGAGAAGCCTATCCCAGTGG
	P. me rpL3 R	GAGCTTTTTAGTGTGCCAACGG
